# The impact of the gut microbiota on the reproductive and metabolic endocrine system

**DOI:** 10.1080/19490976.2021.1894070

**Published:** 2021-03-15

**Authors:** Xinyu Qi, Chuyu Yun, Yanli Pang, Jie Qiao

**Affiliations:** a Center for Reproductive Medicine, Department of Obstetrics and Gynecology, Peking University Third Hospital, Beijing, China; b Key Laboratory of Assisted Reproduction (Peking University), Ministry of Education, Beijing, China; c Key Laboratory of Molecular Cardiovascular Science, Ministry of Education, School of Basic Medical Sciences, Peking University, Beijing, China; d Beijing Key Laboratory of Reproductive Endocrinology and Assisted Reproductive Technology (Peking University Third Hospital), Beijing, China; e National Clinical Research Center for Obstetrics and Gynecology (Peking University Third Hospital), Beijing, China; f Research Units of Comprehensive Diagnosis and Treatment of Oocyte Maturation Arrest, Chinese Academy of Medical Sciences, Beijing, China

**Keywords:** Gut microbiota, reproductive endocrine, estrogen, androgen, insulin

## Abstract

As the gut microbiota exerts various effects on the intestinal milieu which influences distant organs and pathways, it is considered to be a full-fledged endocrine organ. The microbiota plays a major role in the reproductive endocrine system throughout a woman’s lifetime by interacting with estrogen, androgens, insulin, and other hormones. Imbalance of the gut microbiota composition can lead to several diseases and conditions, such as pregnancy complications, adverse pregnancy outcomes, polycystic ovary syndrome (PCOS), endometriosis, and cancer; however, research on the mechanisms is limited. More effort should be concentrated on exploring the potential causes and underlying the mechanisms of microbiota-hormone-mediated disease, and providing novel therapeutic and preventive strategies.As the gut microbiota exerts various effects on the intestinal milieu which influences distant organs and pathways, it is considered to be a full-fledged endocrine organ. The microbiota plays a major role in the reproductive endocrine system throughout a woman’s lifetime by interacting with estrogen, androgens, insulin, and other hormones. Imbalance of the gut microbiota composition can lead to several diseases and conditions, such as pregnancy complications, adverse pregnancy outcomes, polycystic ovary syndrome (PCOS), endometriosis, and cancer; however, research on the mechanisms is limited. More effort should be concentrated on exploring the potential causes and underlying the mechanisms of microbiota-hormone-mediated disease, and providing novel therapeutic and preventive strategies.

## Introduction

The gut microbiota has various effects on the intestinal milieu that influence distant organs and pathways, and the gut microbiota is considered to be a full-fledged endocrine organ. The microbiota plays a major role in the reproductive endocrine system throughout a woman’s lifetime by interacting with estrogens, androgens, insulin, and other hormones. Imbalances in the gut microbiota composition can lead to several diseases and conditions, such as pregnancy complications, adverse pregnancy outcomes, polycystic ovary syndrome (PCOS), endometriosis, and cancer; however, research on these mechanisms is limited. Research efforts should be focused on exploring the potential causes and underlying mechanisms of microbiota hormone-mediated disease and identifying novel therapeutic and preventive strategies.

The gut microbiome contains a large amount of information. It is well known that the number of bacteria in the body is of the same order as the number of human cells^1^ and that the amount of genetic information present in these microbes is at least 150-fold greater than that in the human genome.^2^ Increasing evidence obtained in recent years has suggested that these microorganisms function almost as an extra organ by actively participate in shaping and maintaining our physiology. Numerous host and environmental factors, including diet, host genes, and hormones, are associated with variations in the gut microbiome. Sex hormones, such as progesterone, estradiol, and testosterone, also participate in communication between microorganisms and their hosts and play a number of important physiological roles in reproduction, differentiation, cell proliferation, apoptosis, inflammation, metabolism, homeostasis, and brain function.^3^ Commensal bacteria can produce and secrete hormones, and the crosstalk between microbes and hormones can affect host metabolism, immunity, and behavior. The human microbiome affects every stage and level of female reproduction, including follicle and oocyte maturation in the ovary, fertilization and embryo migration, implantation, and the whole pregnancy, even during parturition. Alterations in the microbiome, particularly the gut microbiome, have specific impacts on the reproductive endocrine system, and correcting abnormal microbiomes may lead to improved reproductive outcomes.^4^ Specific linear correlations between gut microbiota and serum hormone levels, which may have additional effects on the health of the body, have been reported in several studies. Sex hormone levels have a potential relationship with the gut microbiota, and this novel concept has been named the “*microgenderome*”^5^ Moreover, there may be a possible relationship between specific intestinal bacteria and female diseases, such as PCOS, endometriosis and bacterial vaginosis (BV). In this paper, we systematically review the interaction between the gut microbiota and the female endocrine system.

## Interaction of estrogen and the gut microbiome

The gut microbiota is not only influenced by estrogens but also active in affecting estrogen levels. Estrogens are a principal regulator of the gut microbiome, and the gene repertoire of the gut microbiota that is capable of metabolizing estrogens is known as the “estrobolome”^6^

The expression of estrogen receptor β (ERβ) and serum concentrations of steroidal hormones, especially estradiol, are known to change throughout the life cycle of the organism; therefore, regulation of estrogen is essential for women’s health. Intestinal bacteria play an important role in estrogen metabolism, evidenced by the observation that the use of antibiotics leads to lower estrogen levels.^7^ Microbially secreted β-glucuronidase can metabolize estrogens from their conjugate forms to their deconjugated forms.^6^ Dysbiosis and a reduction in gut microbiota diversity reduce β-glucuronidase activity and result in decreased deconjugation of estrogen and phytestrogen into their circulating and active forms. The decrease in circulating estrogens alters estrogen receptor activation and may lead to hypestrogenic pathologies: obesity, metabolic syndrome, cardiovascular disease and cognitive decline.^4,6^ Increased abundance of β-glucuronidase-producing bacteria can lead to elevated levels of circulating estrogens and drive diseases, such as endometriosis and cancer. In addition, estrogen levels can also affect the states of diseases and processes, including PCOS, endometrial hyperplasia, and ultimately fertility.^8^ It was reported that representative orders such as *Lactobacillales* and specific phyla such as *Proteobacteria, Bacteroidetes*, and *Firmicutes* also differ as a function of murine ERβ status, suggesting that steroid nuclear receptor status and dietary complexity may play important roles in microbiota maintenance.^9^ Alpha diversity has a negative correlation with estradiol concentrations, but the mechanism remains unclear. It is possible that gut microbes participate in the regulation of sex hormones and, conversely, that sex hormones modify microbial diversity.^10^

Recently, it was reported that the gut microbiome mediates the preventive effect of 17β-estradiol against metabolic endotoxaemia and low-grade chronic inflammation. 17β-estradiol-treated male and ovariectomized female mice have decreased *Proteobacteria* and lipopolysaccharide (LPS) biosynthesis, and these levels are similar to those of normal female mice. Estrogen or estrogen-like compounds can decrease the LPS produced by the gut microbiome and gut permeability, resulting in reduced metabolic endotoxaemia.^11^ In addition, estrogen can modify gut epithelial barrier integrity in mice, evidenced by the observation that females are more resistant to gut injury than their male counterparts.^12^

Estrogen is also associated with a variety of sex hormone-driven cancers, such as endometrial, cervical, ovarian, prostate and breast cancer. The gut microbiota composition is altered in many of these cancers, and it may play an important role in promoting these cancers.^13^ For example, it was hypothesized that high-fat-diet (HFD)-associated steroids can influence the gastrointestinal microbiome by introducing a carcinogen that might act on breast tissue or an estrogen that might contribute to tumor growth.^14^ Decreased ratios of estrogen metabolites to parental compounds and decreased fecal microbiota diversity are associated with an increased risk of breast cancer in postmenopausal women.^15^

In postmenopausal women, gut microbiota diversity is positively associated with the ratio of estrogen metabolites in urine.^16^ It has been reported that total fat mass and abdominal fat, critical factors in the future development of insulin resistance and type 2 diabetes, are increased in postmenopausal women compared with premenopausal women.^17,18^ The gut microbiota may play an important role in regulating estrogen levels and metabolism during menopause. Furthermore, the gut microbiota can metabolize estrogen-like compounds in foods such as soy isoflavones and promote the growth of some specific bacteria,^19^ and supplementation with soy isoflavones increases the concentration of *Bifidobacterium* and suppresses unclassified *Clostridiaceae* in postmenopausal women.^20,21^ In this regard, *Bifidobacterium* plays a beneficial role in promoting absorption and immunity and preventing infection in the intestine, while *Clostridiaceae* is known to be involved in inflammatory diseases and is associated with obesity.

These results indicate that this host microbe-estrogen interaction contributes to a wide range of pathways that affect women’s health and disease. Estrogens and gut microbiota might synergize to influence various aspects of women’s health, including fertility, obesity, diabetes, and cancer. Therefore, a better understanding of the interactions between estrogens and gut microbiota will lead to new insights and new approaches toward reducing the risk of endocrine diseases in women.

## Interaction between hyperandrogenaemia and intestinal flora

Androgens are also very important hormones in women, and hyperandrogenaemia (HA) is a salient feature of PCOS and a major contributor to hirsutism, acne, male pattern alopecia, and anovulation in affected women. In women with PCOS, elevated luteinizing hormone (LH) leads to the production of excess androgens by ovarian theca cells, and low FSH contributes to impaired folliculogenesis and anovulation, which is the most common reason for infertility secondary to ovulatory dysfunction.^22^ HA conditions have serious health consequences, including increased risks for insulin resistance, type 2 diabetes, hypertension, obesity, and cardiovascular disease. Testosterone may influence the composition of the gut microbiome in females. A regression analysis showed that decreased abundances of several genera were correlated with higher circulating testosterone levels and impaired glucose metabolism in PCOS mice.^10^ Removal of the microbiota increases the circulating testosterone concentration in female mice but decreases the circulating testosterone concentration in male mice, suggesting a bidirectional interaction between the amount of male sex hormone and the microbiota.^23^ Prenatal androgen (PNA) exposure has a powerful effect on a developing female fetus, as daughters of mothers with PCOS are more likely to be diagnosed with PCOS, and transgenerational effects are manifested in both reproductive and metabolic dysfunctions mediated by in utero and/or oocyte-derived factors.^24^ In a dihydrotestosterone (DHT)-induced mouse model, the relative abundance of *Anaerococcus* is enriched in the high androgen group and positively correlated with the levels of testosterone and free testosterone.^25^ The gut microbiota and its metabolites can activate the inflammatory pathway, brain-gut peptide secretion, and islet β-cell proliferation, leading to abnormal or excessive fat accumulation, insulin resistance, and compensatory hyperinsulinaemia.^26,27^

In a testosterone cypionate-induced mouse model, the fecal microbiota profile of PNA animals contained an increased relative abundance of bacteria associated with steroid hormone synthesis and metabolite production of short-chain fatty acids (SCFAs). The cardiovascular function of the mice was also impacted, suggesting that early-life exposure to androgens in female offspring of women with PCOS may lead to long-term alterations in gut microbiota and cardiometabolic function.^28^ *Ruminococcus* has been reported to be significantly increased in neonatally androgenized rats and is positively correlated with serum testosterone levels.^29,30,31^ Shearmen *et al*. reported that there is a negative correlation between alpha diversity and total testosterone, hyperandrogenism, and hirsutism. In animal models of PNA exposure and maternal HA, the relative abundance of bacteria associated with steroid synthesis (*Nocardiaceae* and *Clostridiaceae*) and elongation of unsaturated SCFAs is increased, while that of *Akkermansia, Bacteroides, Lactobacillus*, and *Clostridium* is decreased. These models also had increased body weight and mRNA expression of adipokines, and cardiovascular function was negatively impacted due to increased systolic and diastolic blood pressure and a decreased heart rate.^28^ On the other hand, Insenser *et al*. found a positive correlation between alpha diversity and testosterone levels and the ratio of free testosterone to free estradiol, indicating that gut microbiota may play a role in the regulation of sex hormones and that sex hormones may modify microbial diversity.^10^ However, the limitation of these studies is that there was no in-depth assessment of the mechanistic basis for the interaction between gut microbiota and androgens. A variety of gut microbiota can express the enzymes involved in androgen metabolism and help to synthesize and transform androgens. The degradation of testosterone via microbial processes has been observed in several environmental matrices; for example, *Actinobacteria* and *Proteobacteria* are capable of degrading androgen,^32^ and *Clostridium scindens*, whose genome encodes 20α-hydroxysteroid dehydrogenase (HSDH), is a human gut microbe with high potential to convert glucocorticoids into androgens.^33^

In summary, excess androgen may result in dysbiosis of the host gut microbiota, and changes in the gut microbiome may influence the development and pathology of the endocrine systems of women, even in those with PCOS. Future studies should focus on mechanistic details to provide a comprehensive theoretical basis and therapeutic targets for the diagnosis and treatment of HA.

## Gut microbiota and its role in thyroid disease

The thyroid is a vital endocrine gland. The main function of the thyroid is to secrete the iodine-containing thyroid hormones triiodothyronine (T_3_) and thyroxine (T_4_) and the peptide hormone calcitonin, which affects cardiovascular and reproductive diseases by influencing metabolism and tissue development.^34^

The intestinal microbiota plays a prominent role in thyroid disorders, including Hashimoto’s thyroiditis (HT) and Graves’ disease (GD).^35^ HT and GD are the major causes of hypothyroidism and hyperthyroidism, respectively, and the gut microbiota plays a vital role in thyroid disorders.^36^ It was reported that gut microbial diversity is different between patients with thyroid cancer and healthy control subjects. The abundances of *Neisseria* and *Streptococcus* are markedly elevated in the gut microbiota of patients with thyroid cancer and thyroid nodules, and the abundances of *Butyricimonas* and *Lactobacillus* are reduced.^37^ In patients with HT, some genera of bacteria, such as *Blautia, Roseburia, Ruminococcus_toques_group, Romboustsia, Dorea, Fusicatenibacter*, and *Eubacterium*_*hallii_group*, are increased and correlated with clinical parameters.^38^ In the Graves’ orbitopathy or ophthalmopathy (GO) murine model, the abundance of *Firmicutes* in the intestine is higher and positively correlated with orbital adipogenesis.^39^ The microbiota can also affect the uptake of iodine, selenium, iron, and zinc.^36^ These findings demonstrate that the composition of the gut microbiota can cause and affect the progression of thyroid disorders.

Bile acid, an important metabolite of gut microbiota, plays an essential role in thyroid diseases. The serum bile acid profiles of hyperthyroid and hypothyroid patients are different, and the composition of bile acid can reflect thyroid function.^40^ The most prominent bile acid in hypothyroid patients is the secondary bile acid deoxycholic acid, whereas chenodeoxycholic acid is the most prominent bile acid in hyperthyroid patients. After medical treatment, the levels of bile acid return to normal in patients with thyroid dysfunction. The composition and level of bile acid are involved in the secretion of thyroid hormone, and the thyroid-stimulating hormone level is inversely associated with serum total bile acid in subclinical hypothyroidism.^41^ Thyroid hormone can also suppress bile acid synthesis in primary human hepatocytes.^42^ However, another study demonstrated that thyroid hormone could reduce circulating proprotein convertase subtilisin/kexin type 9 (PSCK9) levels and stimulate bile acid synthesis in humans.^43^ Bile acids can induce energy expenditure and have beneficial metabolic effects by promoting cyclic-AMP-dependent thyroid hormone activating enzyme type 2 iodothyronine deiodinase (D2).^44^

SCFAs interact with the thyroid hormone triiodothyronine and affect the secretion of hormones by regulating enterocyte gene transcription.^45^ For instance, fecal SCFAs can affect women’s emotional health by influencing hyperlipidemia and thyroid disease.^46^

Normal thyroid function is important to maintain healthy reproductive function in both sexes. During pregnancy, both hypothyroidism and hyperthyroidism cause increased rates of spontaneous miscarriage and preterm delivery.^47^ Among women undergoing in vitro fertilization and embryo transfer (IVF-ET), treatment with levothyroxine does not reduce the miscarriage rate among those who test positive for anti-thyroperoxidase antibodies compared to those with normal thyroid function. Thyroid disorders have been demonstrated to be linked to reduced fertility, hypomenorrhea, and polymenorrhea and potentially linked to gestation-induced hypertension and placental abruption.^48^ Given the above data, the gut microbiota may influence the reproductive endocrine system by inducing thyroid dysfunction, such as hypothyroidism or hyperthyroidism.

## Obesity affects intestinal flora and reduces fertility

Obesity and overweight have become pandemic due to HFDs, low energy expenditure, dysbiosis in gut microbiota, or other environmental exposures,^49^ which have adverse effects in terms of disorders of female reproductive endocrine processes. Of note, the role of gut microbiota in the development of obesity has become increasingly important. In an obese mouse model in which T cells disabled the Myd88 pathway, *Clostridia* colonization was reduced.^50^ In mice, treatment against *Akkermansia muciniphila* by means of a purified membrane protein isolated from *A. muciniphila* or the pasteurized bacterium has been shown to prevent the development of obesity and associated complications.^51,52^ In a proof-of-concept exploratory study, supplementation with *A. muciniphila* in overweight and obese humans improved metabolism.^53^ The genus *Bifidobacterium* has also been shown to have a consistent weight loss effect in humans.^54^ These data suggest that the gut microbiota can modify host metabolism and that dysbiotic gut microbiota plays a driving role in the pathogenesis of obesity.

**Figure 1.**
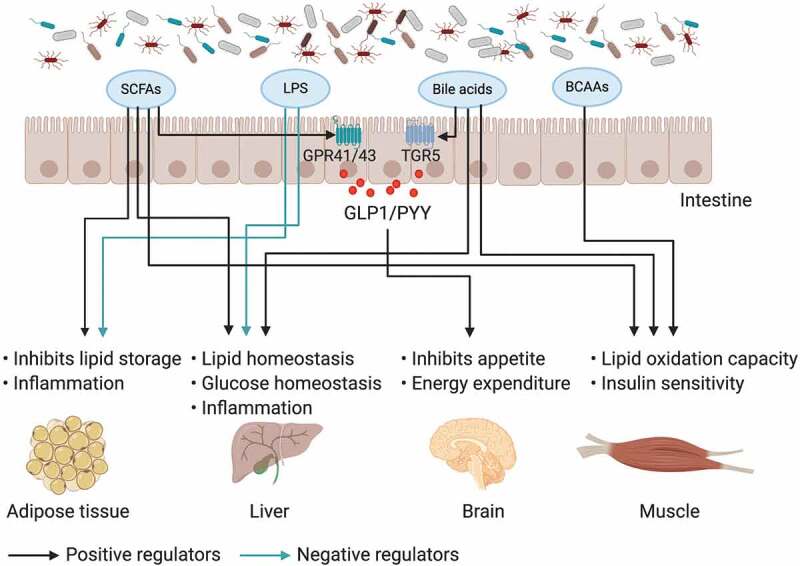
The impact of gut microbiota and its metabolites on inter-organ crosstalk contributes to obesity and insulin resistance

Although the mechanisms by which the gut microbiota regulates host metabolism are incompletely understood, small molecular metabolites derived from gut microbiota have been well investigated in obesity (**Figure 1**).^55–57^ Bile acids are generated from cholesterol in the liver and are metabolized by the gut microbiota in the intestine;^58,59^ additionally, they play a pivotal role in nutrient absorption and biliary secretion of cholesterol.^60^ The decreased levels of the gut microbiome component *Lactobacillus* expressing bile salt hydrolase (BSH) or the direct inhibition of bacterial BSH by caffeic acid phenethyl ester (CAPE) increase the production of tauro-β-muricholic acid (T-β-MCA).^61,62^ Bile acids are involved in obesity, and intestinal increased T-β-MCA, which is a farnesoid X receptor (FXR) nuclear receptor antagonist, improves obesity and hyperglycemia.^61^ TGR5 is another bile acid receptor that can be activated by secondary bile acids and induce cAMP/PKA signaling to increase energy expenditure in brown adipose tissue and muscle and increase the release of glucagon-like peptide 1 (GLP-1) in intestinal L cells.^63^ SCFAs are the main metabolites produced by the bacterial fermentation of indigestible carbohydrates (for example, dietary fiber) and have an important role in the prevention and treatment of obesity.^55,64^ SCFAs stimulate enteroendocrine cells to secrete peptide YY (PYY) and GLP-1 through G protein-coupled receptors (GPR41 and GPR43).^65,66^ SCFAs have beneficial metabolic effects on protection against HFD-induced obesity via a PPARγ-dependent switch from lipogenesis to fat oxidation.^67^ It was also reported that butyrate could improve diet-induced obesity, dyslipidaemia and the development of hepatic steatosis, mainly by reducing appetite and activating brown adipose tissue.^68^ Several human intervention studies also demonstrated that dietary fiber intake decreased body weight and increased the production of satiety-stimulating hormones.^69,70^ Although gut microbial metabolites have been demonstrated to play an important role in obesity-associated complications, further clinical studies are needed.

The gut microbiota and its metabolites communicate with distant organs (host adipose tissue, muscle, liver and brain). SCFAs derived from the fermentation of gut microbiota, LPS, bile acids and BCAAs play important roles in inter-organ crosstalk by regulating gut integrity and influencing peripheral tissue function and metabolism; furthermore, they could contribute to obesity and insulin resistance.

Gut microbiota dysbiosis in obesity is associated with reproductive endocrine diseases. A recent study showed that DHT and HFD shift the overall gut microbial composition and that PCOS rats have a lower Shannon and Simpson index. Moreover, these rats have abnormal estrous cycles with increasing androgen levels and exhibit multiple large ovarian cysts with diminished granulosa layers.^25^ In a clinical study, compared with healthy controls, patients with PCOS showed an imbalance in the intestinal microbiome. With the lower abundance of intestinal SCFAs in PCOS, the abundances of the *Faecalibacterium, Bifidobacterium*, and *Blautia* genera were decreased, while the abundances of *Parabacteroides* and *Clostridium* were increased. Furthermore, the administration of the probiotic *Bifidobacterium* lactis V9 could improve sex hormone levels in PCOS.^71^ Accumulative studies have demonstrated that obesity can disrupt processes related to female fertility, including sex hormone secretion, oocyte differentiation and maturation, and endometrial implantation, and other reproductive functions.^72,73^ Interestingly, bariatric surgery can improve fertility, reduce the risk of pregnancy complications, and improve fetal health.^74,75^ Imbalances in the gut microbiome in obese individuals induce increases in LPS, endotoxaemia, IL-6, and IL-1β, which have proinflammatory functions.^76^ Inflammation reduces oocyte quality, disrupts meiotic and cytoplasmic maturation and is involved in the development of reproductive diseases. Furthermore, the gut microbiota of maternal obesity during pregnancy influences microbiota colonization and metabolism in offspring.^77^ In a Swedish nationwide register-based cohort and a clinical case-control study from Chile, they found that female offspring of mothers who were obese and had elevated androgen levels were more likely to be diagnosed with PCOS, but the role of the gut microbiome remains unclear.^24^

The composition of the gut microbiome and its metabolites in obese individuals is different from that in healthy individuals. The specific microbiota or metabolites derived from the microbiota can influence the pathogenesis of obesity and further cause reproductive endocrine disorders by affecting host metabolism or the inflammatory stage.

## Gut microbiome and its role in insulin resistance

Insulin resistance and compensatory hyperinsulinism contribute to androgen excess in PCOS and other reproductive diseases because insulin can induce androgen secretion from the adrenal glands and regulate the level of luteinizing hormone.^22,78^ Accumulative evidence has demonstrated that the gut microbiome plays an important role in regulating the secretion of insulin.^79^

Insulin is an important hormone that increases membrane permeability to glucose and lowers the level of glucose by activating the insulin receptor. The binding of insulin to insulin receptors causes the activation of insulin receptor tyrosine kinase and the tyrosine phosphorylation of insulin receptor and insulin receptor substrate (IRS) proteins.^80,81^ Phosphorylation of IRS stimulates the binding of the lipid kinase phosphatidylinositol-3-kinase (PI3-K) at the plasma membrane, which phosphorylates the Thr308 residue of AKT by synthesizing Ptdlns(3,4,5)P3 (PIP3).^82^ AKT activation leads to glucose production, utilization and uptake, as well as the synthesis of glycogen, lipids, and proteins.^82^ Interestingly, mounting evidence suggests that the gut microbiome, as well as the metabolites of bacteria, are involved in the progression of insulin resistance.^57,83,84^

A recent study showed that the serum metabolome of insulin-resistant individuals is characterized by increased levels of branched-chain amino acids (BCAAs), which are associated with an increased abundance of the species *Prevotella copri* and *Bacteroides vulgatus* (*B. vulgatus*). *P. copri* can also induce insulin resistance, aggravate glucose intolerance, and increase the levels of BCAAs.^85^ The administration of *Phellinus linteus* polysaccharide extract (PLPE) to mice was found to change the gut microbiota composition and increase SCFA levels, which reduces LPS content and systemic inflammation and improves insulin resistance by inhibiting JNK and NFκB activation.^86^ SCFAs, which are the main fermentation products of the intestinal microbiota and include propionate, acetate, and butyrate, affect metabolic processes of the host, especially insulin resistance.^87^ Human and animal studies have suggested that acetate beneficially affects host metabolism and improves insulin resistance through the gut hormone GLP-1 secreted from colonic L cells, which inhibits appetite and reduces lipolysis and systemic proinflammatory cytokine levels.^88^ Additionally, it was reported that the microbiota-generated metabolites propionate and butyrate activate intestinal gluconeogenesis through complementary mechanisms.^64^

Fecal microbiota transplantation (FMT) is a method of modulating microbial composition and has been widely explored for the treatment of a growing range of microbiome-associated diseases, including insulin resistance and obesity.^89^ Administration of *Collinsella* can reduce the expression of tight junction proteins in intestinal cells and increase the permeability of the intestinal wall. Moreover, *Collinsella* is highly correlated with serum insulin levels, suggesting that the gut microbiota can significantly affect glucose metabolic function.^90^ The transplantation of fecal microbiota from lean donors to obese male recipients with metabolic syndromes improves insulin sensitivity and is accompanied by an altered microbiota composition.^91^

Insulin resistance and compensatory hyperinsulinaemia induce excess androgen and cause reproductive diseases such as PCOS. Insulin can induce the secretion of androgen from the adrenal glands and modulate LH pulsatility.^78,92^ Conversely, hyperandrogenism induces inflammation of abdominal and visceral fat and causes insulin resistance and metabolic dysfunction, thus forming negative feedback in PCOS.^78^ Transcriptional and epigenetic changes in skeletal muscle may promote metabolic abnormalities (such as insulin resistance) in women with PCOS, and the investigators identified differentially expressed genes that were associated with muscle function and metabolism, such as *COL1A1* and *MAP2K6*.^93^ *Bacteroides* is a proinflammatory bacterium that plays a critical role in insulin resistance via an inflammatory mechanism. It was reported that *Bacteroides* was increased in patients with PCOS^25^ and displayed a negative correlation with fasting insulin levels. Administration of IL-22 or glycodeoxycholic acid (GDCA) could help relieve insulin resistance in PCOS mice.^94^

Metformin is widely used for the treatment of individuals with type 2 diabetes (T2D) because of its distinct glucose-lowering effect and safety profile. Recently, it was reported that the gut microbiota plays an important role in the mechanism of action of metformin and that metformin can alter the gut microbiota composition and affect metabolic pathways.^95–97^ The gut microbiome profiles and gut-derived metabolites are related to host insulin sensitivity.^85^ Metformin can increase several SCFA-producing microbiota with increased butyrate and propionate, which are involved in glucose homeostasis, and can improve insulin resistance.^98^ Sun *et al*. further investigated the role of microbiota and its metabolites in the hypoglycemic effect of metformin and its detailed molecular mechanism. They found that the species *Bacteroides fragilis* was decreased and that bile acid glycoursodeoxycholic acid (GUDCA) was increased in the gut after metformin treatment, with concomitant inhibition of intestinal FXR signaling. Briefly, metformin improves metabolic dysfunction in part through the *B. fragilis*–GUDCA–intestinal FXR axis.^99^ However, Ara Koh *et al*. reported that the microbial metabolite imidazole propionate impairs the glucose-lowering effect of metformin through p38γ-dependent inhibitory AMPK phosphorylation.^100^

Overall, these studies indicate that the microbiota and its metabolites are involved in the pathogenesis of insulin resistance (**Figure 1**). Insulin resistance is one of the symptoms of female reproductive diseases such as PCOS, which has been demonstrated to cause gut microbiota dysfunction; however, the underlying mechanisms of microbe-insulin resistant reproductive diseases remain to be further studied.

## Microbiota, the female reproductive tract, and embryo development

**Figure 2.**
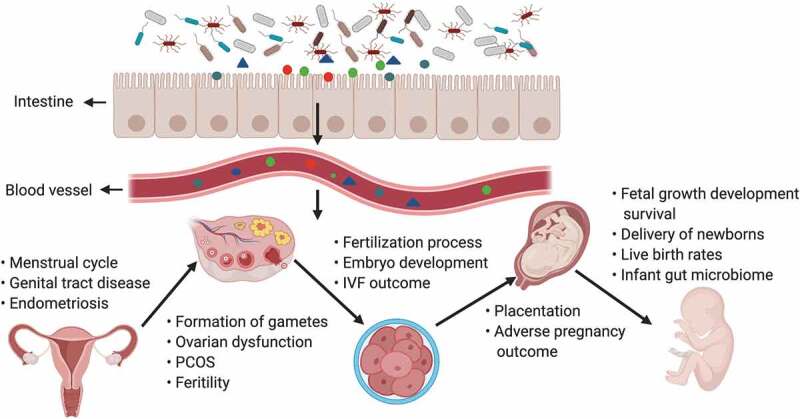
The gut microbiota and its impact on the female reproductive tract, embryo development and pregnancy

The gut microbiota and its products may influence all of embryonic development from the formation of gametes to the processes involved in fertilization, implantation of the conceptus, placentation, abortion, delivery of the new-borns, and metabolic programming and reprogramming during critical periods of the life cycle (**Figure 2**). According to reports, many bacteria present in the digestive tract are also present in the female reproductive tract, including the vagina, endometrium and placenta.^101–103^ Functionally, the microbiota is a continuum in the reproductive tract and can be perturbed at multiple sites in a disease.

Products of gut microbiota may be transported through the circulation and influence the female reproductive tract (e.g., may cause disruptions in the menstrual cycle, genital tract disease and endometriosis), ovarian function, embryonic development and the health of the mother and fetus. The gut can affect the formation of gametes, embryo development and fertilization processes, and alteration of intestinal flora can lead to ovarian dysfunction, PCOS, infertility, and adverse IVF outcomes. In pregnant women, the gut microbiota has an effect on placentation, pregnancy outcomes, the delivery of new-born babies, the infant microbiome, the live birth rate and fetal growth, development, and survival.

The vaginal microbiome is dynamic and seems to be affected by the menstrual cycle, the dominant microbiota, and sexual activity.^104^ Women who deliver preterm exhibit significantly lower vaginal species levels of *Lactobacillus crispatus* and higher levels of *Sneathia amnii*, which are correlated with proinflammatory cytokines in vaginal fluid.^105^ A meta-analysis including five independent studies across 3,201 samples demonstrated that there are significant differences between the microbial within-sample variance among women who delivered prematurely compared to those who delivered at term. Moreover, the differences were observed across pregnancy trimesters and are consistent across different racial or ethnic backgrounds.^106^ The maternal vaginal microbiome can alter the composition of the microbiota colonizing the neonatal gut. Microbiota diversity, structure, and composition in non-inoculated cesarean-delivered offspring are significantly decreased compared with those in vaginally delivered offspring.^107^ Prenatal stress is also associated with alterations in the fetal intestinal transcriptome and niche, as well as with changes in the adult gut that occur due to additional exposure to stress in adulthood.^107^

Recently, the molecular identification of bacterial species in the endometrium confirmed that the uterine cavity is not sterile.^108^ Furthermore, endometrial communities also arise from colonization of specific macroflora, with a number of *Bacteroidetes* and *Proteobacteria* taxa primarily associated with the gastrointestinal tract.^109^ There are several differing opinions suggesting that the presence of uterine microbiota is likely reflective of bacterial tourists or invaders rather than a resident population that contributes to health and homeostasis.^110^ The presence of bacterial species in the uterine cavity for patients undergoing in vitro fertilization (IVF) has negative effects on the implantation and pregnancy rates.^111^ The presence of a non-*Lactobacillus*-dominated microbiota in a receptive endometrium is associated with significant decreases in implantation, pregnancy, ongoing pregnancy, and live birth rates,^102^ but the pathogens and the mechanisms by which they interfere with embryonic implantation remain unclear. The intestinal microbiome and its metabolites can affect endometrial and uterine immunity during implantation and placentation as a possible interaction partner of the local microbiota.^112^ For example, T cells are a large fraction of immune cells in the human endometrium, and a proper ratio of Th1 and Th2 cells within the endometrium is needed to prepare for implantation. The intestinal microbiome and metabolites can provide immunostimulatory signals and activate innate and downstream adaptive immune responses, and the differentiation of various T cell subsets is impaired in mice with disrupted gut microbiota.^113,114^ Because T cells reside mostly in the deeper layers of the endometrium, they are more likely to have a role in early placenta formation after implantation.

Establishment and maintenance of placental integrity and function are critical for fetal growth, development, and survival. Whether the placenta has its own colonized flora remains controversial. A recent report demonstrated that there was no evidence of the presence of bacteria in the large majority of placental samples from both complicated and uncomplicated pregnancies. However, in approximately 5% of pregnant women, there is an important pathogen, *S. agalactiae*, in the placenta before the onset of labor.^115^ Another study indicated that the placenta harbors a low-abundance but metabolically rich microbiome, and variations in the placental microbiome are associated with a remote history of antenatal infection, which may have a profound influence on intrauterine infection and preterm birth.^103^ According to one recent study of new-borns, the genus *Escherichia* has a high abundance in meconium and is a strong contributor to early-onset sepsis among extremely low-birth-weight neonates, and the placenta is a likely source of *Escherichia* in the new-born meconium.^116^ In a recent study, SCFAs in the bloodstream were able to pass from a non-germ-free mother’s gut microbiota across the placenta and into developing embryos. Embryonic insulin regulation was impaired in embryos from germ-free (GF) mothers, and insulin levels were significantly elevated in the adult stage, providing evidence for the crucial contribution of the maternal gut environment to the metabolic programming of offspring.^117^ Patients with preeclampsia showed reduced bacterial diversity with obvious dysbiosis. In the placenta of both patients and mice with preeclampsia, the total bacteria, *Fusobacterium* genus, and inflammatory cytokine levels were significantly increased, suggesting that the gut microbiome is symbiotic and contributes to disease pathogenesis.^118^

Intestinal microflora disorder can lead to ovarian dysfunction, including oocyte developmental disorder, disruption of the estrous cycle, and abnormal ovulation. Changes in the gut bacteria of Drosophila can affect its germ line. Removal of the gut bacteria represses oogenesis, expedites the maternal-to-zygotic transition in the offspring and unmasks hidden phenotypic variation in mutants, and the main impact on oogenesis is linked to the lack of the gut *Acetobacter* species.^119^ Antibiotic amoxicillin may induce gut dysbiosis and alter the estrous cycles of mice, and the oocyte area from the dysbiotic group was lower than that from nondysbiotic mice with increased thickness of the pellucid zone.^120^ Fecal transplantation from women with ovarian disorders to mice leads to ovarian dysfunction and impaired fertility,^94^ and treating PCOS rats with *Lactobacillus* and fecal microbiota from healthy rats leads to recovery of the estrous cycle, with decreasing androgen biosynthesis and normal ovarian morphologies.^121^ Obesity-dependent gut dysbiosis is a contributing factor to the development of ovarian dysfunction associated with metabolic syndrome.^122^ Obesity negatively impacts ovarian function and oocyte quality, and bariatric surgery in obese women can help to normalize ovulatory patterns, improve conception rates and fetal health, reduce pregnancy complications, and improve metabolic function. All of these improvements may be due to changes in the composition of the gut microbiome.^76,123^ The levels of oocyte-specific marker transcripts and proinflammatory signals are significantly higher in the ovaries of obese mice than in the ovaries of lean mice. In addition, there is a positive correlation between the relative ovarian abundance of oocyte-specific transcripts and the cecum microbiota composition in obese mice, indicating that the gut microbiome in obese mice may contribute to reduced oocyte quality.^124^ The microbiome in follicular fluid influences the IVF outcome, and the presence of some microorganisms, especially *Lactobacillus spp*. is associated with better embryo quality and higher rates of embryo transfer and pregnancy.^125^ A recent report demonstrated that the levels of trimethylamine N-oxide (TMAO) and its gut-derived precursor gamma-butyrobetaine are lower in follicular fluid from normally fertilized oocytes that develop into top-quality embryos than in those that do not. These results indicate that the microbiota may play an important role in embryo development and that microbiota-dependent metabolites can be significant predictive biomarkers in the future.^126^

Overall, the gut microbiota is closely related to female reproductive tract health, and the impetus of these studies in the future will be to identify targets that can be used to therapeutically improve the fertility of women with reproductive endocrine disorders.

## Changes in the microbiota and host hormone levels during pregnancy

The composition of the gut microbiota shifts among the different stages of pregnancy, particularly in the third trimester, and these changes lead to differences in metabolic, immunological and hormonal changes, which are necessary and highly beneficial to support a healthy pregnancy and fetal development.^127^ In turn, dramatic shifts in hormone levels, such as those of estrogen and progesterone, also impact gut function and bacterial composition, accompanied by unique inflammatory and immune changes. For example, problems associated with infection by *Listeria monocytogenes* in pregnancy are partly due to elevated estrogen and progesterone levels, leading to adverse outcomes, including preterm delivery or stillbirth.^128^ Progesterone, the principal gestational hormone, affects the contents of several bacterial species, such as the relative abundance of the *Bifidobacterium* genus, which is increased; progesterone facilitates the health of the pregnant mother and is perhaps also helpful to the transmission of beneficial species to the neonate.^129^

The microbial diversity in the gut at the start of pregnancy appears to be similar to that in nonpregnant women, and the abundance of gut bacteria associated with inflammatory states increases as pregnancy advances.^130^ A high abundance of the family *Ruminococcaceae* in early pregnancy may be related to adverse metabolic health, and a high abundance of the families *Lachnospiraceae, Prevotellaceae*, and *Bacteroidaceae* is associated with maternal energy metabolism.^131^ A high abundance of species in the *Coriobacteriaceae* family and *Collinsella* genus may serve as markers of impaired glucose metabolism.^131^ During the third trimester, the abundances of *Proteobacteria* and *Actinobacteria*, especially the *Enterobacteriaceae* family and *Streptococcus* genus, increase substantially, and the gut microbiota may induce greater adiposity and insulin resistance in the mother as they promote energy storage and provide for the growth of fetal metabolism. In addition, compared to the first trimester, the third trimester has elevated low-grade inflammation and reduced oral glucose tolerance, and these disorders can be transferred to GF mice.^127^ The relative abundances of the *Bacteroides* and *Staphylococcus* genera are higher in overweight pregnant women during gestation. The two genera are also associated with enhanced accretion of white adipose tissue in pregnant women and the fetus, indicating that the gut microbiota may regulate maternal and fetal whole-body energy metabolism.^132,133^

The gut microbiome may regulate the stability of maternal metabolism by influencing SCFAs, inflammatory cytokines, bile acid metabolism, and metabolic hormones, leading to the occurrence of diabetes and obesity regardless of pregnancy.^134^ The metabolites of the gut microbiome, including bile acids and SCFAs, have a distinctive position among maternal microbial metabolites that affect the fetus because they can penetrate the placental barrier and are critical to ensuring organ function for development, growth, and immunity.^135^

The metabolic hormone levels (insulin, C-peptide, glucagon, incretin, and adipokine), and microbiome profiles of women who are overweight or obese differ, indicating that metabolic hormones participate in the process in which gut microbiota influences metabolic health during early pregnancy in women who were overweight or obese.^131^ Indeed, the gut microbiota plays an important role in the development of gestational diabetes mellitus (GDM), and the differences in the microbiota between women with GDM and healthy pregnant women are correlated with blood glucose levels;^136^ pregnant women with GDM have gut microbiota dysbiosis, and the gut plays an important role in the development of GDM.^134^

Progesterone and estrogen levels rise dramatically during pregnancy, with significant immunity-related changes, and these are likely to affect the microbiota. Elevated concentrations of progesterone during pregnancy can inhibit the development of Th1 responses and the production of proinflammatory cytokines, such as IFN-γ, and promote Th2 immune responses, including the synthesis of anti-inflammatory cytokines such as IL-4, IL-5, and IL-10.^137^ On the other hand, the maternal microbiota have been shown to shape the offspring’s immune system in terms of immunity-related gene expression and the numbers of cells involved in innate immunity, and it may be of great importance for preventing allergic disease in the offspring.^138^

Due to the changed hormone levels, the vaginal microbiome is also changed significantly during pregnancy, with decreased overall diversity, increased stability, and increased abundance of the *Lactobacillus* species, but these changes occur mainly in early pregnancy; the communities at the later stages resemble those of the nonpregnant state.^139^ Some vaginal communities in early pregnancy stages are associated with preterm birth.^140^

Obesity and hyperglycemia during pregnancy can affect the risk of adverse pregnancy outcomes and the establishment of early gut microbiota in infants, thus affecting the long-term health of mothers and offspring. The most recent belief is that the maternal microbiota shapes the phenotypes of offspring. The infant gut microbiome may be influenced by the maternal microbiome through vertical transfer of maternal microbes to infants during vaginal delivery and breastfeeding.^141^ The maternal diet dramatically alters the intestinal microbiome of mothers and their offspring at one year of age, and a HFD results in significant variation in intestinal taxa among dams with metabolic disorders in a primate study.^142^ Compared to control offspring, offspring from GF mothers are highly susceptible to metabolic syndrome characterized by an exacerbation of obesity and glucose intolerance.^117^ The colonization of microbes in mothers is of great importance to babies during early life and plays a critical role in the establishment and maturation of developmental pathways.^143^ In a mouse model, maternal microbial metabolites drive the development of the pups’ innate immune system and maturation of the intestinal epithelium.^144^

In short, pregnancy is an important and modifying physiological period for women of reproductive age, and the gut microbiome exhibits significant changes during pregnancy. Reconstruction of the gut may change the states of the mother’s endocrine, metabolic, and immune systems, which are necessary for a successful pregnancy (**Figure 2**).

## Reproductive endocrine disorders related to gut microbiota

### PCOS

PCOS is one of the most common reproductive endocrine and metabolic disorders and is defined as a combination of signs and symptoms of excess androgens (hirsutism and/or hyperandrogenaemia) and ovarian dysfunction (anovulation, oligo-ovulation and/or polycystic ovarian morphology). The prevalence of PCOS among women of reproductive age ranges from 6% to 20%.^78^ Patients with PCOS usually have insulin resistance, obesity, low-grade inflammation, and cardiovascular risk factors.^78^ Due to the complex etiology and its heterogeneous nature, the cause of PCOS is unknown, but studies suggest that PCOS might be a complex multigenic disorder with strong epigenetic and environmental influences, including diet and other lifestyle issues.^22,145^ Recent studies have demonstrated that gut microbes play an important role in the etiology of PCOS. In 2012, Pearce *et al*. proposed an assumption that disturbances in bowel bacterial flora (“Dysbiosis of Gut Microbiota”) brought about by a poor diet create an increase in gut mucosal permeability, releasing LPS into the systemic circulation, activating the immune system and driving up serum insulin levels, leading to increased androgen production in the ovary and interference with normal follicular development.^122^ In recent years, many papers clarifying the relationship between the gut microbiota and PCOS have been published and have shed light on the pathogenesis and therapy of this disease. Our previous study showed that the beta diversity of microbiomes in women with PCOS was significantly decreased compared with that in healthy controls, and *B. vulgatus* was markedly increased in PCOS patients. Transplantation of fecal microbiota from women with PCOS or *B. vulgatus*-colonized recipient mice resulted in PCOS-like mice, accompanied by decreased IL-22 and GDCA, indicating that the gut microbiota–bile acid–interleukin-22 axis regulates PCOS.^94^ Another study showed that PCOS mice had decreased abundances of gut microbiome species and decreased phylogenetic diversity and that *Firmicutes* levels were significantly higher in PCOS mice than in control mice.^146^ The abundance of the *Tenericutes* phylum in women with PCOS was significantly lower than that in healthy women.^147,148^ In addition, there was a decrease in *Akkermensia* and *Ruminococcaceae*, and gram-negative bacteria belonging to the *Bacteroides* and *Escherichia/Shigella* genera were significantly increased in PCOS women, especially women who were obese. Under this condition, LPS produced by gram-negative bacteria was demonstrated to induce chronic inflammation, obesity, and insulin resistance in LPS-infused mice. Compared with controls, PCOS patients have significant decreases in the serotonin, ghrelin, and PYY levels, indicating that the mediators of the brain-gut axis are associated with PCOS.^149^ A recent study showed that compared with that of BMI-matched normal adolescents, the gut microbiome of obese adolescents with PCOS is altered and that these alterations include decreased alpha diversity, which is strongly associated with higher testosterone concentrations.^150^

Treating PCOS rats with the *Lactobacillus* genus and FMT from healthy rats can improve estrous cycles and ovarian morphologies and decrease androgen biosynthesis, indicating that improving the structure of the intestinal flora can relieve the symptoms of PCOS.^121^ In two randomized, double-blind, placebo-controlled trials, probiotic supplementation for women with PCOS had favorable effects on weight loss, insulin resistance, triglycerides and VLDL cholesterol concentration,^151,152^ and probiotics also helped improve total testosterone, SHBG and proinflammatory cytokine production.^152^ Another clinical trial found that coadministration of probiotics and selenium had beneficial effects on mental health parameters, serum total testosterone levels, hirsutism, and biomarkers of inflammation and oxidative stress, such as hs-CRP, the total antioxidant capacity, and total glutathione, in women with PCOS, indicating that probiotics can modulate inflammation in women with PCOS.^153^

Metformin is one of the most effective drugs used in the treatment of type 2 diabetes and insulin resistance and has been shown to improve fertility outcomes among females with insulin resistance associated with PCOS. It can help to reinstate menstrual cyclicity, improve hyperandrogenism and ovulation, decrease the incidence of cesarean sections, and limit the number of premature births.^154,155^ However, the exact mechanism by which metformin helps to improve PCOS remains unclear, though the gut microbiome may play an important role. In a recent study, inflammatory indicators from the plasma and ovary, including TNF-α, IL-6, and IL-17A, were decreased in a metformin-treated PCOS mouse model compared with the control group, and the *Helicobacter* genus was decreased in the metformin-treated group. Metformin can alleviate PCOS via its anti-inflammation properties and modulation of the gut microbiota, which may contribute to potential clinical therapy for the disease.^156^

This offers a new therapeutic strategy for the clinical diagnosis and treatment of PCOS.

## Endometriosis

Endometriosis is a condition defined by the presence of endometrial glands and stroma outside of the uterine cavity; in this condition, dysbiosis influences estrogen levels in circulation, and increased estrogen levels can stimulate the growth of ectopic endometriotic foci and inflammatory activity.^157^ Endometriosis is considered a typical multifactorial condition that may be determined by genetic, immunological, and environmental factors. Because gut flora affects estrogen metabolism, inflammation and homeostasis, recent studies have shown that gut microbiota may be closely involved in the onset and progression of endometriosis. Because the microbiota is involved in the regulation of estrogen cycling, gut dysbiosis increases the levels of circulating estrogen, which may markedly stimulate the growth and cyclic bleeding of endometriotic lesions. On the other hand, a number of immune cell subtypes and inflammatory factors are altered in endometriosis, as are inflammatory cytokine levels in the peritoneal fluid and serum, which are increased, reflecting dysregulation and alterations in the gut microbiome and gut permeability.^158^ The gut microbiota plays an important role in the systemic inflammatory response and activation of immune cells, and imbalances in the intestinal flora are thought to play a role in the pathogenesis of endometriosis. Once the balance between estrogen levels in circulation and the gut microbiome is disrupted, increased estrogen exposure can stimulate the development and progression of endometriosis.^159^ There is a 50% increase in the risk of inflammatory bowel disease in women with endometriosis, indicating a strong interaction between immunological processes in the gut and endometriotic lesions.^160^ In a murine model of endometriosis, the *Firmicutes*/*Bacteroidetes* ratio was elevated, and *Bifidobacterium* was also increased.^161^ In another study, endometriotic lesions and inflammatory responses in mice treated with broad-spectrum antibiotics were significantly reduced compared to those in control mice. Oral gavage of feces from mice with endometriosis restored endometriotic lesion growth and inflammation, suggesting that gut bacteria promote endometriosis progression in mice.^162^ Endometriosis is associated with lower *lactobacilli* concentrations and more gram-negative bacteria as well as a higher prevalence of intestinal inflammation in rhesus monkeys, while the mechanisms linking these effects remain unclear.^163^ A recent study showed that the vaginal, cervical and gut microbiota compositions were similar between women with stage 3–4 endometriosis and controls, but patients with endometriosis had more *Escherichia*/*Shigella* in their stool.^159^

Taken together, these observations indicate there may be a direct link between pathological changes in the gut microbiota and the onset and progression of endometriosis. Thus, there is an urgent need to characterize the complex pathophysiological condition of endometriosis, to establish the basis for the development of novel diagnostic and therapeutic strategies.

## Female genital tract diseases

The vaginal microbiota typically changes throughout the menstrual cycle, and microbiota stability is associated with the estradiol peak at ovulation and the progesterone rise in the midluteal phase. However, alterations in the vaginal microbiota can lead to several pathologies. BV is a dysbiosis of the vaginal microbiome that has a close relationship with significant adverse healthcare outcomes, including an increased risk of abnormal pregnancy outcomes, pelvic inflammatory disease, and increased susceptibility to sexually transmitted infections.^164^ BV is characterized by low levels of “healthy” *lactobacilli* and overgrowth of numerous bacteria, including *Gardnerella, Atopobium, Mobiluncus, Prevotella, Bacteroides, Sneathia, Leptotrichia*, and members of the Clostridia class, among others.^165,166^

The most prevalent vaginal *Lactobacillus* species have been shown to colonize the rectum, and cocolonization of the vagina and rectum correlates with the lowest prevalence of BV.^167^ The rectum is a key reservoir for vaginal lactobacilli, and rectal colonization with these microorganisms contributes to the maintenance of health-associated vaginal microbiota; furthermore, gut microbiota can indirectly influence genital microbiota through the estrobolome.^168^ A meta-analysis that included 12 studies comprising a total of 2980 patients showed that tubal factor infertility was significantly more prevalent in IVF patients diagnosed with BV than in patients without BV and that BV was significantly associated with early spontaneous abortion in IVF patients.^169^ The promotion of *Lactobacillus* colonization with probiotics, modulation of vaginal pH, hormonal administration, and eradication of pathogenic bacteria with antibiotics could alter the cervicovaginal microbiota and have beneficial effects on women with BV.^170^

There is strong evidence that intestinal-vaginal interactions play a key role in BV. The challenge is to find tools and strategies capable of modulating microbial populations in the cervicovaginal microenvironment; understanding the connection between intestinal and vaginal microbiota may be a goal for new treatments of female genital tract disorders.

## Conclusion and future perspectives

In conclusion, the unique dynamic ecosystem of the gastrointestinal microbiota humans acquire in early life is regarded as a metabolically active “organ”, which is responsible for the health of the individual and development of diseases throughout life. Steroids and metabolic hormones play vital roles in intestinal homeostasis, and dysbiotic microbiota may trigger intestinal barrier dysfunction and affect other organs, leading to chronic low-grade inflammation, metabolic disorders, and immune dysfunction. Together, estrogens, androgens, progestogen, insulin, and other hormones regulate women’s health from the womb to the tomb. Although studies have shown that the gut microbiota has a vital function in the female reproductive endocrine system, further mechanistic studies are still needed. In the future, extensive and functional studies will make it possible to use the gut microbiota as a biomarker for certain diseases, and manipulating the gut microbiota in specific and directed ways will serve to move the field forward. By using probiotics or fecal transplantation, we will be able to treat dysbiosis and prevent disease development.

## References

[1] Sender R, Fuchs S, Milo R. Revised estimates for the number of human and bacteria cells in the body[J]. PLoS Biol. 2016; 14(8): e1002533. doi:10.1371/journal.pbio.1002533 27541692 PMC4991899

[2] Qin J, Li R, Raes J, Arumugam M, Burgdorf KS, Manichanh C, Nielsen T, Pons N, Levenez F, Yamada T, et al. A human gut microbial gene catalogue established by metagenomic sequencing[J]. Nature. 2010; 464(7285):59–21. doi:10.1038/nature08821 20203603 PMC3779803

[3] Edwards DP. Regulation of signal transduction pathways by estrogen and progesterone[J]. Annu Rev Physiol. 2005; 67(1): 335–376. doi:10.1146/annurev.physiol.67.040403.120151 15709962

[4] Franasiak JM, Scott RT. Introduction: microbiome in human reproduction[J]. Fertil Steril. 2015; 104(6): 1341–1343. doi:10.1016/j.fertnstert.2015.10.021 26515381

[5] M B F, J F N, Blumberg RS. Immunology. Welcome to the microgenderome[J] Science. 2013;339:1044–1045. 23449586 10.1126/science.1236226 PMC4005781

[6] Plottel C, Blaser MJ. Microbiome and malignancy[J]. Cell Host Microbe. 2011; 10(4): 324–335. doi:10.1016/j.chom.2011.10.003 22018233 PMC3264051

[7] Adlercreutz H, Pulkkinen MO, E.k. H, Korpela JT. Studies on the role of intestinal bacteria in metabolism of synthetic and natural steroid hormones[J]. J Steroid Biochem. 1984; 20(1): 217–229. doi:10.1016/0022-4731(84)90208-5 6231418

[8] J M B, Al-Nakkash L, Herbst-Kralovetz MM. Estrogen-gut microbiome axis: physiological and clinical implications[J]. Maturitas. 2017; 103:45–53. 28778332 10.1016/j.maturitas.2017.06.025

[9] Menon R, Watson SE, Thomas LN, Allred CD, Dabney A, Azcarate-Peril MA, Sturino JM. Diet complexity and estrogen receptor beta status affect the composition of the murine intestinal microbiota[J]. Appl Environ Microbiol. 2013; 79(18):5763–5773. 23872567 10.1128/AEM.01182-13 PMC3754184

[10] Insenser M, Murri M, Campo RD, Martínez-García MA, Fernández-Durán E, Escobar-Morreale HF. Gut microbiota and the polycystic ovary syndrome: influence of sex, sex hormones, and obesity[J]. J Clin Endocrinol Metab. 2018; 103(7):2552–2562. 29897462 10.1210/jc.2017-02799

[11] Kaliannan K, Robertson RC, Murphy K, Stanton C, Kang C, Wang B, Hao L, Bhan AK , Kang JX. Estrogen-mediated gut microbiome alterations influence sexual dimorphism in metabolic syndrome in mice[J]. Microbiome. 2018; 6(1):205. 30424806 10.1186/s40168-018-0587-0 PMC6234624

[12] Homma H, Hoy E, Xu DZ, Lu Q, Feinman R, Deitch EA. The female intestine is more resistant than the male intestine to gut injury and inflammation when subjected to conditions associated with shock states[J]. Am J Physiol Gastrointest Liver Physiol. 2005; 288(3):G466–G472. 15499084 10.1152/ajpgi.00036.2004

[13] R F S, Jobin C. The microbiome and cancer[J]. Nat Rev Cancer. 2013;13:800–812. 24132111 10.1038/nrc3610 PMC3986062

[14] M J H, Goddard P, Williams RE. Gut bacteria and aetiology of cancer of the breast[J]. Lancet. 1971;2:472–473. 4105334 10.1016/s0140-6736(71)92634-1

[15] Flores R, Shi J, Fuhrman B, Xu X, Veenstra TD, Gail MH, Gajer P, Ravel J, Goedert JJ. Fecal microbial determinants of fecal and systemic estrogens and estrogen metabolites: a cross-sectional study[J]. J Transl Med. 2012; 10:253. 23259758 10.1186/1479-5876-10-253 PMC3552825

[16] Fuhrman B J, Feigelson H S, Flores R, Gail M H, Xu X, Ravel J, Goedert JJ. Associations of the fecal microbiome with urinary estrogens and estrogen metabolites in postmenopausal women[J]. J Clin Endocrinol Metab. 2014; 99(12):4632–4640. 25211668 10.1210/jc.2014-2222 PMC4255131

[17] Lobo R A, Davis S R, Villiers T J D,Gompel A, Henderson V W, Hodis H N, Lumsden M A, Mack W J, Shapiro S, Baber R J. Prevention of diseases after menopause[J]. Climacteric. 2014; 17(5):540–556. 24969415 10.3109/13697137.2014.933411

[18] Leeners B, Geary N, Tobler P N, Asarian L. Ovarian hormones and obesity[J]. Hum Reprod Update. 2017; 23(3):300–321. 28333235 10.1093/humupd/dmw045 PMC5850121

[19] K L C, Estrogen M-EZ. Microbiota crosstalk: should we pay attention?[J]. Trends Endocrinol Metab. 2016;27:752–755. 27553057 10.1016/j.tem.2016.08.001

[20] Frankenfeld C L, Atkinson C, Wahala K, Lampe J W. Obesity prevalence in relation to gut microbial environments capable of producing equol or O-desmethylangolensin from the isoflavone daidzein[J]. Eur J Clin Nutr. 2014; 68(4):526–530. 24569543 10.1038/ejcn.2014.23 PMC4189015

[21] Nakatsu C H, Armstrong A, Clavijo A P, Martin B R, Barnes S, Weaver C M. Fecal bacterial community changes associated with isoflavone metabolites in postmenopausal women after soy bar consumption[J]. PLoS One. 2014; 9(10):e108924. 25271941 10.1371/journal.pone.0108924 PMC4182758

[22] Azziz R, Carmina E, Chen Z, Dunaif A, Laven J S E, Legro R S, Lizneva D ,Natterson-Horowtiz B, Teede H J, Yildiz B O. Polycystic ovary syndrome[J]. Nat Rev Dis Primers. 2016; 2:16057. 27510637 10.1038/nrdp.2016.57

[23] JMarkle J G, Frank D N, Mortin-Toth S, Robertson C E, Feazel L M, Rolle-Kampczyk U, Bergen M V, McCoy K D, Macpherson A J, Danska J S. Sex differences in the gut microbiome drive hormone-dependent regulation of autoimmunity[J]. Science. 2013; 339(6123):1084–1088. 23328391 10.1126/science.1233521

[24] Risal S, Pei Y, Lu H, Manti M, Fornes R , Pui H P , Zhao Z Y , Massart J , Ohlsson C , Lindgren E. Prenatal androgen exposure and transgenerational susceptibility to polycystic ovary syndrome[J]. Nat Med. 2019; 25(12):1894–1904. 31792459 10.1038/s41591-019-0666-1

[25] Zheng Y, Yu J, Liang C, Shuna Li S , Wen X , Li Y. Characterization on gut microbiome of PCOS rats and its further design by shifts in high-fat diet and dihydrotestosterone induction in PCOS rats[J]. Bioprocess Biosyst Eng. 2020;2020 Mar 10. doi: 10.1007/s00449-020-02320-w. 10.1007/s00449-020-02320-w 32157446

[26] Vrieze A, Holleman F, Zoetendal E G, Vos W M D, Hoekstra J B L, Nieuwdorp M. The environment within: how gut microbiota may influence metabolism and body composition[J]. Diabetologia. 2010; 53(4):606–613. 20101384 10.1007/s00125-010-1662-7 PMC2830587

[27] Barber T M, Dimitriadis G K, Andreou A, Franks S. Polycystic ovary syndrome: insight into pathogenesis and a common association with insulin resistance[J]. Clin Med (Lond). 2016; 16(3):262–266. 27251917 10.7861/clinmedicine.16-3-262 PMC5922706

[28] Sherman S B, Sarsour N, Salehi M, Schroering A, Mell B, Joe B, Hill J W. Prenatal androgen exposure causes hypertension and gut microbiota dysbiosis[J]. Gut Microbes. 2018; 9(5):400–421. 29469650 10.1080/19490976.2018.1441664 PMC6219642

[29] Shin J H, Park Y H, Sim M, Kim S A, Joung H, Shin D M. Serum level of sex steroid hormone is associated with diversity and profiles of human gut microbiome[J]. Res Microbiol. 2019; 170(4–5):192–201. 30940469 10.1016/j.resmic.2019.03.003

[30] Moreno-Indias I, Sanchez-Alcoholado L, Sanchez-Garrido M A, Martín-Núñez G M, Pérez-Jiménez F, Tena-Sempere M, Tinahones F J, Queipo-Ortuño M I. Neonatal androgen exposure causes persistent gut microbiota dysbiosis related to metabolic disease in adult female rats[J]. Endocrinology. 2016; 157(12):4888–4898. 27700135 10.1210/en.2016-1317

[31] Sherman S B, Sarsour N, Salehi M, Schroering A, Mell B, Joe B, Hill J W. Prenatal androgen exposure causes hypertension and gut microbiota dysbiosis[J]. Gut Microbes. 2018; 9(5):400–421. 29469650 10.1080/19490976.2018.1441664 PMC6219642

[32] Yang Y Y, Pereyra L P, Young R B, Reardon K F, Borch T. Testosterone-mineralizing culture enriched from swine manure: characterization of degradation pathways and microbial community composition[J]. Environ Sci Technol. 2011; 45(16):6879–6886. 21740029 10.1021/es2013648

[33] Ridlon J M, Ikegawa S, Alves J M, Zhou B, Kobayashi A, Iida T, Mitamura K, Tanabe G, Serrano M, Guzman A D, et al. Clostridium scindens: a human gut microbe with a high potential to convert glucocorticoids into androgens[J]. J Lipid Res. 2013; 54(9):2437–2449. 23772041 10.1194/jlr.M038869 PMC3735941

[34] Roelfsema F, Veldhuis JD. Thyrotropin secretion patterns in health and disease[J]. Endocr Rev. 2013;34:619–657. 23575764 10.1210/er.2012-1076

[35] Young VB. The intestinal microbiota in health and disease[J]. Curr Opin Gastroenterol. 2012;28:63–69. 22080827 10.1097/MOG.0b013e32834d61e9 PMC3707308

[36] Frohlich E, Wahl R. Microbiota and thyroid interaction in health and disease[J]. Trends Endocrinol Metab. 2019;30:479–490. 31257166 10.1016/j.tem.2019.05.008

[37] Zhang J, Zhang F, Zhao C, Xu Q, Liang C, Yang Y, Wang H, Shang Y, Wang Y, Mu X, et al. Dysbiosis of the gut microbiome is associated with thyroid cancer and thyroid nodules and correlated with clinical index of thyroid function[J]. Endocrine. 2019; 64(3):564–574. 30584647 10.1007/s12020-018-1831-x

[38] Zhao F, Feng J, Li J, Zhao L , Liu Y , Chen H , Jin Y , Zhu B , Wei Y. Alterations of the gut microbiota in hashimoto’s thyroiditis patients[J]. Thyroid. 2018; 28(2):175–186. 29320965 10.1089/thy.2017.0395

[39] Masetti G, Moshkelgosha S, Kohling H L, Covelli D, Banga J P, Berchner-Pfannschmidt U, Horstmann M, Diaz-Cano S, Goertz G E, Plum S, et al. Gut microbiota in experimental murine model of Graves’ orbitopathy established in different environments may modulate clinical presentation of disease[J]. Microbiome. 2018; 6(1):97. 29801507 10.1186/s40168-018-0478-4 PMC5970527

[40] Kosuge T, Beppu T, Kodama T, Hidai K, Idezuki Y. Serum bile acid profile in thyroid dysfunction and effect of medical treatment[J]. Clin Sci (Lond). 1987; 73(4):425–429. 3665361 10.1042/cs0730425

[41] Song Y, Zhao M, Zhang H, Zhang X, Zhao J, Xu J, Gao L. THYROID-STIMULATING HORMONE LEVELS ARE INVERSELY ASSOCIATED WITH SERUM TOTAL BILE ACID LEVELS: a CROSS-SECTIONAL STUDY[J]. Endocr Pract. 2016; 22(4):420–426. 26606535 10.4158/EP15844.OR

[42] Ellis EC. Suppression of bile acid synthesis by thyroid hormone in primary human hepatocytes[J]. World J Gastroenterol. 2006;12:4640–4645. 16937432 10.3748/wjg.v12.i29.4640 PMC4087826

[43] Bonde Y, Breuer O, Lutjohann D, Sjöberg S, Angelin B, Rudling M. Thyroid hormone reduces PCSK9 and stimulates bile acid synthesis in humans[J]. J Lipid Res. 2014; 55(11):2408–2415. 25172631 10.1194/jlr.M051664 PMC4617142

[44] Watanabe M, S M H, Mataki C, Christoffolete M A, Kim B W, Sato H, Messaddeq N, Harney J W, Ezaki O, Kodama T, et al. Bile acids induce energy expenditure by promoting intracellular thyroid hormone activation[J]. Nature. 2006; 439(7075):484–489. 16400329 10.1038/nature04330

[45] Meng S, Wu J T, Archer S Y, Hodin R A. Short-chain fatty acids and thyroid hormone interact in regulating enterocyte gene transcription[J]. Surgery. 1999; 126(2):293–298. 10455897

[46] Skonieczna-Zydecka K, Grochans E, Maciejewska D, Szkup M , Schneider-Matyka D , Jurczak A , Łoniewski I , Kaczmarczyk M , Marlicz W , Czerwińska-Rogowska M , et al. Faecal short chain fatty acids profile is changed in polish depressive women[J]. Nutrients. 2018; 10(12):1939. 10.3390/nu10121939 PMC6316414 30544489

[47] Stagnaro-Green A, Pearce E. Thyroid disorders in pregnancy[J]. Nat Rev Endocrinol. 2012;8:650–658. 23007317 10.1038/nrendo.2012.171

[48] G E K, Poppe K, Glinoer D. Thyroid function and human reproductive health[J]. Endocr Rev. 2010;31:702–755. 20573783 10.1210/er.2009-0041

[49] P W F, McCarthy MI. Exposing the exposures responsible for type 2 diabetes and obesity[J]. Science. 2016;354:69–73. 27846494 10.1126/science.aaf5094

[50] Petersen C, Bell R, Klag K A , Lee S H , Soto R , Ghazaryan A , Buhrke K , Ekiz H A , Ost K S , Boudina S , et al. T cell-mediated regulation of the microbiota protects against obesity[J]. Science. 2019; 365(6451):eaat9351. 10.1126/science.aat9351 PMC7294966 31346040

[51] Everard A, Belzer C, Geurts L, Ouwerkerk J P, Druart C, Bindels L B, Guiot Y, Derrien M, Muccioli G G, Delzenne N M, et al. Cross-talk between Akkermansia muciniphila and intestinal epithelium controls diet-induced obesity[J]. Proc Natl Acad Sci U S A. 2013; 110(22):9066–9071. 23671105 10.1073/pnas.1219451110 PMC3670398

[52] Plovier H, Everard A, Druart C, Depommier C, Hul M V, Geurts L, Chilloux J, Ottman N, Duparc T, Lichtenstein L, et al. A purified membrane protein from Akkermansia muciniphila or the pasteurized bacterium improves metabolism in obese and diabetic mice[J]. Nat Med. 2017; 23(1):107–113. 27892954 10.1038/nm.4236

[53] Depommier C, Everard A, Druart C, Plovier H, Hul M V, Vieira-Silva S, Falony G, Raes J, Maiter D, Delzenne N M , et al. Supplementation with Akkermansia muciniphila in overweight and obese human volunteers: a proof-of-concept exploratory study[J]. Nat Med. 2019; 25(7):1096–1103. 31263284 10.1038/s41591-019-0495-2 PMC6699990

[54] Angelakis E, Armougom F, Million M, Raoult D. The relationship between gut microbiota and weight gain in humans[J]. Future Microbiol. 2012; 7(1):91–109. 22191449 10.2217/fmb.11.142

[55] Canfora E E, Meex R, Venema K, Blaak E E. Gut microbial metabolites in obesity, NAFLD and T2DM[J]. Nat Rev Endocrinol. 2019; 15(5):261–273. 30670819 10.1038/s41574-019-0156-z

[56] Zhao L, Zhang F, Ding X, Wu G, Lam Y Y, Wang X, Fu H, Xue X, Lu C, Ma J, et al. Gut bacteria selectively promoted by dietary fibers alleviate type 2 diabetes[J]. Science. 2018; 359(6380):1151–1156. 29590046 10.1126/science.aao5774

[57] T R A, Haeusler RA. Bile acids in glucose metabolism and insulin signalling - mechanisms and research needs[J]. Nat Rev Endocrinol. 2019;15:701–712. 31616073 10.1038/s41574-019-0266-7 PMC6918475

[58] Wahlstrom A, Sayin S I, Marschall H U, Backhed F. Intestinal crosstalk between bile acids and microbiota and its impact on host metabolism[J]. Cell Metab. 2016; 24(1):41–50. 27320064 10.1016/j.cmet.2016.05.005

[59] Jia W, Xie G, Jia W. Bile acid-microbiota crosstalk in gastrointestinal inflammation and carcinogenesis[J]. Nat Rev Gastroenterol Hepatol. 2018;15:111–128. 29018272 10.1038/nrgastro.2017.119 PMC5899973

[60] Chiang JY. Recent advances in understanding bile acid homeostasis[J]. F1000Res. 2017; 6:2029. 29188025 10.12688/f1000research.12449.1 PMC5698910

[61] Li F, Jiang C, Krausz K W, Li Y, Albert I, Hao H,Fabre K M, Mitchell J B, Patterson A D, Gonzalez F J. Microbiome remodelling leads to inhibition of intestinal farnesoid X receptor signalling and decreased obesity[J]. Nat Commun. 2013; 4:2384. 24064762 10.1038/ncomms3384 PMC6595219

[62] Xie C, Jiang C, Shi J, Gao X, Sun D, Sun L, Wang T, Takahashi S, Anitha M, Krausz K W, et al. An intestinal farnesoid x receptor-ceramide signaling axis modulates hepatic gluconeogenesis in mice[J]. Diabetes. 2017; 66(3):613–626. 28223344 10.2337/db16-0663 PMC5319721

[63] Thomas C, Gioiello A, Noriega L, Strehle A, Oury J, Rizzo G, Macchiarulo A, Yamamoto H, Mataki C, Pruzanski M, et al. TGR5-mediated bile acid sensing controls glucose homeostasis[J]. Cell Metab. 2009; 10(3):167–177. 19723493 10.1016/j.cmet.2009.08.001 PMC2739652

[64] De Vadder F, Kovatcheva-Datchary P, Goncalves D, Vinera J, Zitoun C, Duchampt A, Bäckhed F, Mithieux G. Microbiota-generated metabolites promote metabolic benefits via gut-brain neural circuits[J]. Cell. 2014; 156(1–2):84–96. 24412651 10.1016/j.cell.2013.12.016

[65] Tolhurst G, Heffron H, Lam Y S, Parker H E, Habib A M, Diakogiannaki E, Cameron J, Grosse J, Reimann F, Gribble F M. Short-chain fatty acids stimulate glucagon-like peptide-1 secretion via the G-protein-coupled receptor FFAR2[J]. Diabetes. 2012; 61(2):364–371. 22190648 10.2337/db11-1019 PMC3266401

[66] Psichas A, Sleeth M L, Murphy K G, Brooks L, Bewick G A, Hanyaloglu A C, Ghatei M A , Bloom S R, Frost G. The short chain fatty acid propionate stimulates GLP-1 and PYY secretion via free fatty acid receptor 2 in rodents[J]. Int J Obes (Lond). 2015; 39(3):424–429. 25109781 10.1038/ijo.2014.153 PMC4356745

[67] den Besten G, Bleeker A, Gerding A, Eunen K V, Havinga R, Dijk T H V, Oosterveer M H, Jonker J W, Groen A K, Reijngoud D J, et al. Short-chain fatty acids protect against high-fat diet-induced obesity via a ppargamma-dependent switch from lipogenesis to fat oxidation[J]. Diabetes. 2015; 64(7):2398–2408. 25695945 10.2337/db14-1213

[68] Li Z , Yi C X, Katiraei S, Kooijman S, Zhou E, Chung C K, Gao Y, Heuvel J K V D, Meijer O C, Berbée J F P, et al. Butyrate reduces appetite and activates brown adipose tissue via the gut-brain neural circuit[J]. Gut. 2018; 67(7):1269–1279. 29101261 10.1136/gutjnl-2017-314050

[69] Thompson S V, Hannon B A, An R, Holscher H D. Effects of isolated soluble fiber supplementation on body weight, glycemia, and insulinemia in adults with overweight and obesity: a systematic review and meta-analysis of randomized controlled trials[J]. Am J Clin Nutr. 2017; 106(6):1514–1528. 29092878 10.3945/ajcn.117.163246

[70] J A P, Reimer RA. Weight loss during oligofructose supplementation is associated with decreased ghrelin and increased peptide YY in overweight and obese adults[J]. Am J Clin Nutr. 2009;89:1751–1759. 19386741 10.3945/ajcn.2009.27465 PMC3827013

[71] Zhang J, Sun Z, Jiang S, Bai X, Ma C, Peng Q, Chen K, Chang H, Fang T, Zhang H. Probiotic bifidobacterium lactis v9 regulates the secretion of sex hormones in polycystic ovary syndrome patients through the gut-brain axis[J]. mSystems. 2019; 4(2):e00017-19. 10.1128/mSystems.00017-19 PMC6469956 31020040

[72] Silvestris E, de Pergola G, Rosania R, Loverro G. Obesity as disruptor of the female fertility[J]. Reprod Biol Endocrinol. 2018; 16(1):22. 29523133 10.1186/s12958-018-0336-z PMC5845358

[73] Chavarro J E, Rich-Edwards J W, Rosner B A, Willett W C. Diet and lifestyle in the prevention of ovulatory disorder infertility[J]. Obstet Gynecol. 2007; 110(5):1050–1058. 17978119 10.1097/01.AOG.0000287293.25465.e1

[74] Guelinckx I, Devlieger R, Vansant G. Reproductive outcome after bariatric surgery: a critical review[J]. Hum Reprod Update. 2009;15:189–201. 19136457 10.1093/humupd/dmn057

[75] Gosman GG, King WC, Schrope B, Steffen K J, Strain G W, Courcoulas A P, Flum D R, Pender J R , Simhan H N. Reproductive health of women electing bariatric surgery[J]. Fertil Steril. 2010; 94(4):1426–1431. 19815190 10.1016/j.fertnstert.2009.08.028 PMC2888936

[76] A P S, Wood JR. Obesity induces ovarian inflammation and reduces oocyte quality[J]. Reproduction. 2019;158:R79–R90. 30999278 10.1530/REP-18-0583

[77] Zhou L, Xiao X. The role of gut microbiota in the effects of maternal obesity during pregnancy on offspring metabolism[J]. Biosci Rep. 2018; 38(2):BSR20171234. 10.1042/BSR20171234 PMC5897743 29208770

[78] Escobar-Morreale HF. Polycystic ovary syndrome: definition, aetiology, diagnosis and treatment[J]. Nat Rev Endocrinol. 2018;14:270–284. 29569621 10.1038/nrendo.2018.24

[79] A M C, Saad MJ. The role of gut microbiota on insulin resistance[J]. Nutrients. 2013;5:829–851. 23482058 10.3390/nu5030829 PMC3705322

[80] Kasuga M, Zick Y, Blithe D L, Kahn C R. Insulin stimulates tyrosine phosphorylation of the insulin receptor in a cell-free system[J]. Nature. 1982; 298(5875):667–669. 6178977 10.1038/298667a0

[81] Sun X J, Rothenberg P, Kahn C R, Backer J M, Araki E, Wilden P A, Cahill D A, Goldstein B J, White M F. Structure of the insulin receptor substrate IRS-1 defines a unique signal transduction protein[J]. Nature. 1991; 352(6330):73–77. 1648180 10.1038/352073a0

[82] R A H, T E M, Accili D. Biochemical and cellular properties of insulin receptor signalling[J]. Nat Rev Mol Cell Biol. 2018;19:31–44. 28974775 10.1038/nrm.2017.89 PMC5894887

[83] Jiang C, Xie C, Lv Y, Li J, Krausz K W, Shi J, Brocker C N, Desai D, Amin S G, Bisson W H, et al. Intestine-selective farnesoid X receptor inhibition improves obesity-related metabolic dysfunction[J]. Nat Commun. 2015; 6:10166. 26670557 10.1038/ncomms10166 PMC4682112

[84] A C G, Hoffmann C, Mota JF. The human gut microbiota: metabolism and perspective in obesity[J]. Gut Microbes. 2018;9:308–325. 29667480 10.1080/19490976.2018.1465157 PMC6219651

[85] Pedersen H K, Gudmundsdottir V, Nielsen H B, Hyotylainen T, Nielsen T, Jensen B A H, Forslund K, Hildebrand F, Prifti E, Falony G, et al. Human gut microbes impact host serum metabolome and insulin sensitivity[J]. Nature. 2016; 535(7612):376–381. 27409811 10.1038/nature18646

[86] Liu Y, Wang C, Li J, Li T , Zhang Y , Liang Y , Mei Y. Phellinus linteus polysaccharide extract improves insulin resistance by regulating gut microbiota composition[J]. Faseb J. 2020; 34(1):1065–1078. 31914668 10.1096/fj.201901943RR

[87] C J L, C L S, Maruthur N. Gut microbiome and its role in obesity and insulin resistance[J]. Ann N Y Acad Sci. 2020;1461:37–52. 31087391 10.1111/nyas.14107

[88] Hernandez M, Canfora E E, Jocken J, Blaak E E. The short-chain fatty acid acetate in body weight control and insulin sensitivity[J]. Nutrients. 2019; 11(8):1943. 10.3390/nu11081943 PMC6723943 31426593

[89] Olesen S W, Panchal P, Chen J, Budree S , Osman M. Global disparities in faecal microbiota transplantation research[J]. Lancet Gastroenterol Hepatol. 2020; 5(3):241. 32061326 10.1016/S2468-1253(19)30452-2

[90] Chen J, Wright K, Davis J M, Jeraldo P, Marietta E V, Murray J, Nelson H, Matteson E L, Taneja V. An expansion of rare lineage intestinal microbes characterizes rheumatoid arthritis[J]. Genome Med. 2016; 8(1):43. 27102666 10.1186/s13073-016-0299-7 PMC4840970

[91] R S K, Levin E, Salojarvi J, Smits L P, Hartstra A V, Udayappan S D, Hermes G, Bouter K E, Koopen A M , Holst J J. Improvement of insulin sensitivity after lean donor feces in metabolic syndrome is driven by baseline intestinal microbiota composition[J]. Cell Metab. 2017; 26(4):611–619. 28978426 10.1016/j.cmet.2017.09.008

[92] Nestler J E, Jakubowicz D J, de Vargas A F, Brik C, Quintero N, Medina F. Insulin stimulates testosterone biosynthesis by human thecal cells from women with polycystic ovary syndrome by activating its own receptor and using inositolglycan mediators as the signal transduction system[J]. J Clin Endocrinol Metab. 1998; 83(6):2001–2005. 9626131 10.1210/jcem.83.6.4886

[93] Nilsson E, Benrick A, Kokosar M,, Krook A, Lindgren E, Källman T, Martis M M, Højlund K, Ling C, Stener-Victorin E. Transcriptional and epigenetic changes influencing skeletal muscle metabolism in women with polycystic ovary syndrome[J]. J Clin Endocrinol Metab. 2018; 103(12):4465–4477. 30113663 10.1210/jc.2018-00935

[94] Qi X, Yun C, Sun L, Xia J, Wu Q, Wang Y, Wang L, Zhang Y, Liang X, Wang L, et al. Gut microbiota-bile acid-interleukin-22 axis orchestrates polycystic ovary syndrome[J]. Nat Med. 2019; 25(8):1225–1233. 31332392 10.1038/s41591-019-0509-0 PMC7376369

[95] Wu H, Esteve E, Tremaroli V, Khan M T, Caesar R, Mannerås-Holm L, Ståhlman M, Olsson L M, Serino M, Planas-Fèlix M, et al. Metformin alters the gut microbiome of individuals with treatment-naive type 2 diabetes, contributing to the therapeutic effects of the drug[J]. Nat Med. 2017; 23(7):850–858. 28530702 10.1038/nm.4345

[96] N G V, Stratigou T, Tsagarakis S. Metformin and gut microbiota: their interactions and their impact on diabetes[J]. Hormones (Athens). 2019;18:141–144. 30719628 10.1007/s42000-019-00093-w

[97] Lee H, Lee Y, Kim J, An J, Lee S, Kong H, Song Y, Lee C K, Kim K. Modulation of the gut microbiota by metformin improves metabolic profiles in aged obese mice[J]. Gut Microbes. 2018; 9(2):155–165. 29157127 10.1080/19490976.2017.1405209 PMC5989809

[98] Perry R J, Peng L, Barry N A, Cline G W, Zhang D, Cardone R L, Petersen K F, Kibbey R G, Goodman A L, Shulman G I. Acetate mediates a microbiome-brain-beta-cell axis to promote metabolic syndrome[J]. Nature. 2016; 534(7606):213–217. 27279214 10.1038/nature18309 PMC4922538

[99] Sun L, Xie C, Wang G, Wu Y , Wu Q , Wang X , Liu J , Deng Y , Xia J , Chen B , et al. Gut microbiota and intestinal FXR mediate the clinical benefits of metformin[J]. Nat Med. 2018; 24(12):1919–1929. 30397356 10.1038/s41591-018-0222-4 PMC6479226

[100] Koh A, Manneras-Holm L, Yunn N O, Nilsson P M, Ryu S H, Molinaro A, Perkins R,Smith J G, Bäckhed F. Microbial imidazole propionate affects responses to metformin through p38gamma-dependent inhibitory ampk phosphorylation[J]. Cell Metab. 2020;Oct 6;32(4):643-653.e4. 10.1016/j.cmet.2020.07.012 PMC7546034 32783890

[101] Gajer P, R M B, Bai G, Sakamoto J, Schütte U M E, Zhong X, Koenig S S K, Fu L, Ma Z S, Zhou X, et al. Temporal dynamics of the human vaginal microbiota[J]. Sci Transl Med. 2012; 4(132):132r–152r. 10.1126/scitranslmed.3003605 PMC3722878 22553250

[102] Moreno I, Codoñer F M, Vilella F, Valbuena D, Martinez-Blanch J F, Jimenez-Almazán J, Alonso R, Alamá P, Remohí J, Pellicer A, et al. Evidence that the endometrial microbiota has an effect on implantation success or failure[J]. Am J Obstet Gynecol. 2016; 215(6):684–703. 27717732 10.1016/j.ajog.2016.09.075

[103] Aagaard K, Ma J ,Antony K M ,Ganu R, Petrosino J, Versalovic J. The placenta harbors a unique microbiome[J]. Sci Transl Med. 2014; 6(237):237r–265r. 10.1126/scitranslmed.3008599 PMC4929217 24848255

[104] K A G, S M Z, Catherino WH. Gynecologic health and disease in relation to the microbiome of the female reproductive tract[J]. Fertil Steril. 2015;104:1351–1357. 26597627 10.1016/j.fertnstert.2015.10.010

[105] Fettweis J M, Serrano M G, Brooks J P, Edwards D J, Girerd P H, Parikh H I, Huang B , Arodz T J, Edupuganti L, Glascock A L, et al. The vaginal microbiome and preterm birth[J]. Nat Med. 2019; 25(6):1012–1021. 31142849 10.1038/s41591-019-0450-2 PMC6750801

[106] Kosti I, Lyalina S, Pollard K S, Butte A J, Sirota M. Meta-analysis of vaginal microbiome data provides new insights into preterm birth[J]. Front Microbiol. 2020; 11:476. 32322240 10.3389/fmicb.2020.00476 PMC7156768

[107] Jasarevic E, Howard C D, Morrison K, Misic A, Weinkopff T, Scott P, Hunter C, Beiting D, Bale T L. The maternal vaginal microbiome partially mediates the effects of prenatal stress on offspring gut and hypothalamus[J]. Nat Neurosci. 2018; 21(8):1061–1071. 29988069 10.1038/s41593-018-0182-5

[108] Moller B R, Kristiansen F V, Thorsen P, Frost L, Mogensen S C. Sterility of the uterine cavity[J]. Acta Obstet Gynecol Scand. 1995; 74(3):216–219. 7900526 10.3109/00016349509008942

[109] Verstraelen H, Vilchez-Vargas R, Desimpel F,, Jauregui R, Vankeirsbilck N, Weyers S, Verhelst R, Sutter P D, Pieper D H, Wiele T V D. Characterisation of the human uterine microbiome in non-pregnant women through deep sequencing of the V1-2 region of the 16S rRNA gene[J]. PeerJ. 2016; 4:e1602. 26823997 10.7717/peerj.1602 PMC4730988

[110] J M B, D M C, Herbst-Kralovetz MM. Uterine Microbiota: residents, Tourists, or Invaders?[J]. Front Immunol. 2018; 9:208. 29552006 10.3389/fimmu.2018.00208 PMC5840171

[111] Romero R, Chaiworapongsa T, Kuivaniemi H, Tromp G. Bacterial vaginosis, the inflammatory response and the risk of preterm birth: a role for genetic epidemiology in the prevention of preterm birth[J]. Am J Obstet Gynecol. 2004; 190(6):1509–1519. 15284723 10.1016/j.ajog.2004.01.002

[112] Benner M, Ferwerda G, Joosten I, van der Molen RG. How uterine microbiota might be responsible for a receptive, fertile endometrium[J]. Hum Reprod Update. 2018; 24(4):393–415. 29668899 10.1093/humupd/dmy012

[113] Omenetti S, Pizarro TT. The Treg/Th17 axis: a dynamic balance regulated by the gut microbiome[J]. Front Immunol. 2015; 6:639. 26734006 10.3389/fimmu.2015.00639 PMC4681807

[114] Gaboriau-Routhiau V, Rakotobe S, Lecuyer E, Mulder I, Lan A, Bridonneau C, Rochet V, Pisi A, De Paepe M, Brandi G, et al. The key role of segmented filamentous bacteria in the coordinated maturation of gut helper T cell responses[J]. Immunity. 2009; 31(4):677–689. 19833089 10.1016/j.immuni.2009.08.020

[115] de Goffau M C, Lager S, Sovio U, Gaccioli F, Cook E, Peacock SJ, Parkhill J, Charnock-Jones DS, Smith GCS. Human placenta has no microbiome but can contain potential pathogens[J]. Nature. 2019; 572(7769):329–334. 31367035 10.1038/s41586-019-1451-5 PMC6697540

[116] Gosalbes MJ, Llop S, Vallès Y, Moya A, Ballester F, Francino MP. Meconium microbiota types dominated by lactic acid or enteric bacteria are differentially associated with maternal eczema and respiratory problems in infants[J]. Clin Exp Allergy. 2013; 43(2):198–211. 23331561 10.1111/cea.12063

[117] Kimura I, Miyamoto J, Ohue-Kitano R, Watanabe K, Yamada T, Onuki M, Aoki R, Isobe Y, Kashihara D, Inoue D, et al. Maternal gut microbiota in pregnancy influences offspring metabolic phenotype in mice[J]. Science. 2020; 367:6481. 10.1126/science.aaw8429 32108090

[118] Chen X, Li P, Liu M, Zheng H, He Y, Chen MX, Tang W, Yue X, Huang Y, Zhuang L, et al. Gut dysbiosis induces the development of pre-eclampsia through bacterial translocation[J]. Gut. 2020; 69(3):513–522. 31900289 10.1136/gutjnl-2019-319101

[119] Elgart M, Stern S, Salton O, Gnainsky Y, Heifetz Y, Soen Y. Impact of gut microbiota on the fly’s germ line[J]. Nat Commun. 2016; 7:11280. 27080728 10.1038/ncomms11280 PMC4835552

[120] Silva EN, Martins TVF, Miyauchi-Tavares TM, Miranda BAE, Dos Santos GA, Rosa CP, Santos JA, Novaes RD, de Almeida LA, Corsetti PP. Amoxicillin-induced gut dysbiosis influences estrous cycle in mice and cytokine expression in the ovary and the caecum[J]. Am J Reprod Immunol. 2020;84(1): e13247. 32304259 10.1111/aji.13247

[121] Guo Y, Qi Y, Yang X, Zhao L, Wen S, Liu Y, Tang L. Association between polycystic ovary syndrome and gut microbiota[J]. PLoS One. 2016; 11(4):e153196. 10.1371/journal.pone.0153196 PMC4836746 27093642

[122] Tremellen K, Pearce K. Dysbiosis of gut microbiota (DOGMA)–a novel theory for the development of polycystic ovarian syndrome[J]. Med Hypotheses. 2012;79:104–112. 22543078 10.1016/j.mehy.2012.04.016

[123] Guelinckx I, Devlieger R, Vansant G. Reproductive outcome after bariatric surgery: a critical review[J]. Hum Reprod Update. 2009;15:189–201. 19136457 10.1093/humupd/dmn057

[124] Xie F, Anderson CL, Timme KR, Kurz SG, Fernando SC, Wood JR. Obesity-dependent increases in oocyte mRNAs are associated with increases in proinflammatory signaling and gut microbial abundance of lachnospiraceae in female mice[J]. Endocrinology. 2016; 157(4):1630–1643. 26881311 10.1210/en.2015-1851 PMC4816731

[125] Pelzer ES, Allan JA, Waterhouse MA,, Ross T, Beagley KW, Knox CL. Microorganisms within human follicular fluid: effects on IVF[J]. PLoS One. 2013; 8(3):e59062. 23554970 10.1371/journal.pone.0059062 PMC3595219

[126] Nagy RA, Homminga I, Jia C, Liu F, Anderson JLC, Hoek A, Tietge UJF. Trimethylamine-N-oxide is present in human follicular fluid and is a negative predictor of embryo quality[J]. Hum Reprod. 2020; 35(1):81–88. 31916569 10.1093/humrep/dez224 PMC9185935

[127] Koren O, Goodrich J K, Cullender T C, Spor A, Laitinen K, Bäckhed H K, Gonzalez A, Werner J J, Angenent L T, Knight R, et al. Host remodeling of the gut microbiome and metabolic changes during pregnancy[J]. Cell. 2012; 150(3):470–480. 22863002 10.1016/j.cell.2012.07.008 PMC3505857

[128] Garcia-Gomez E, Gonzalez-Pedrajo B, Camacho-Arroyo I. Role of sex steroid hormones in bacterial-host interactions[J]. Biomed Res Int. 2013; 2013:928290. 23509808 10.1155/2013/928290 PMC3591248

[129] Nuriel-Ohayon M, Neuman H, Ziv O, Belogolovski A, Barsheshet Y, Bloch N, Uzan A, Lahav R, Peretz A, Frishman S, et al. Progesterone increases bifidobacterium relative abundance during late pregnancy[J]. Cell Rep. 2019; 27(3):730–736. 30995472 10.1016/j.celrep.2019.03.075

[130] Edwards S M, Cunningham S A, Dunlop A L, Corwin E J. The maternal gut microbiome during pregnancy[J]. MCN Am J Matern Child Nurs. 2017; 42(6):310–317. 28787280 10.1097/NMC.0000000000000372 PMC5648614

[131] Gomez-Arango L F, Barrett H L, McIntyre H D, Callaway L K, Morrison M, Dekker Nitert M; SPRING Trial Group. Connections between the gut microbiome and metabolic hormones in early pregnancy in overweight and obese women[J]. Diabetes. 2016; 65(8):2214–2223. 27217482 10.2337/db16-0278

[132] Collado MC, Isolauri E, Laitinen K, Salminen S. Distinct composition of gut microbiota during pregnancy in overweight and normal-weight women[J]. Am J Clin Nutr. 2008; 88(4):894–899. 18842773 10.1093/ajcn/88.4.894

[133] Santacruz A, Collado MC, García-Valdés L, Segura MT, Martín-Lagos JA, Anjos T, Martí-Romero M, Lopez RM, Florido J, Campoy C, et al. Gut microbiota composition is associated with body weight, weight gain and biochemical parameters in pregnant women[J]. Br J Nutr. 2010; 104(1):83–92. 20205964 10.1017/S0007114510000176

[134] Lv Y, Yan Z, Zhao X, Gang X, He G, Sun L, Li Z, Wang G. The effects of gut microbiota on metabolic outcomes in pregnant women and their offspring[J]. Food Funct. 2018; 9(9):4537–4547. 30101246 10.1039/c8fo00601f

[135] S C G-V, M W H, Macpherson AJ. Microbial-host molecular exchange and its functional consequences in early mammalian life[J]. Science. 2020;368:604–607. 32381716 10.1126/science.aba0478

[136] Kuang YS, Lu JH, Li SH, Li JH, Yuan MY, He JR, Chen NN, Xiao WQ, Shen SY, Qiu L, et al. Connections between the human gut microbiome and gestational diabetes mellitus[J]. Gigascience. 2017; 6(8):1–12. 10.1093/gigascience/gix058 PMC5597849 28873967

[137] D P R, Klein SL. Pregnancy and pregnancy-associated hormones alter immune responses and disease pathogenesis[J]. Horm Behav. 2012;62:263–271. 22406114 10.1016/j.yhbeh.2012.02.023 PMC3376705

[138] Gomez de Agüero M, Ganal-Vonarburg SC, Fuhrer T, Rupp S, Uchimura Y, Li H, Steinert A, Heikenwalder M, Hapfelmeier S, Sauer U, et al. The maternal microbiota drives early postnatal innate immune development[J]. Science. 2016; 351(6279):1296–1302. 26989247 10.1126/science.aad2571

[139] Aagaard K, Riehle K, Ma J, Segata N, Mistretta TA, Coarfa C, Raza S, Rosenbaum S, Van den Veyver I, Milosavljevic A, et al. A metagenomic approach to characterization of the vaginal microbiome signature in pregnancy[J]. PLoS One. 2012; 7(6):e36466. 22719832 10.1371/journal.pone.0036466 PMC3374618

[140] DiGiulio DB, Callahan BJ, McMurdie PJ, Costello EK, Lyell DJ, Robaczewska A, Sun CL, Goltsman DS, Wong RJ, Shaw G, et al. Temporal and spatial variation of the human microbiota during pregnancy[J]. Proc Natl Acad Sci U S A. 2015; 112(35):11060–11065. 26283357 10.1073/pnas.1502875112 PMC4568272

[141] Lundgren SN, Madan JC, Emond JA, Morrison HG, Christensen BC, Karagas MR, Hoen AG. Maternal diet during pregnancy is related with the infant stool microbiome in a delivery mode-dependent manner[J]. Microbiome. 2018; 6(1):109. 29973274 10.1186/s40168-018-0490-8 PMC6033232

[142] Ma J, Prince AL, Bader D, Hu M, Ganu R, Baquero K, Blundell P, Alan Harris R, Frias AE, Grove KL,et al. High-fat maternal diet during pregnancy persistently alters the offspring microbiome in a primate model[J]. Nat Commun. 2014; 5:3889. 24846660 10.1038/ncomms4889 PMC4078997

[143] Robertson RC, Manges AR, Finlay BB, Prendergast AJ. The human microbiome and child growth - first 1000 days and beyond[J]. Trends Microbiol. 2019; 27(2):131–147. 30529020 10.1016/j.tim.2018.09.008

[144] Gomez de Agüero M, Ganal-Vonarburg SC, Fuhrer T, Rupp S, Uchimura Y, Li H, Steinert A, Heikenwalder M, Hapfelmeier S, Sauer U, et al. The maternal microbiota drives early postnatal innate immune development[J]. Science. 2016; 351(6279):1296–1302. 26989247 10.1126/science.aad2571

[145] Norman RJ, Dewailly D, Legro RS, Hickey TE. Polycystic ovary syndrome[J]. Lancet. 2007; 370(9588):685–697. 17720020 10.1016/S0140-6736(07)61345-2

[146] Kelley S T, Skarra DV, Rivera A J, Thackray VG. The gut microbiome is altered in a letrozole-induced mouse model of polycystic ovary syndrome[J]. PLoS One. 2016; 11(1):e146509. 10.1371/journal.pone.0146509 PMC4701222 26731268

[147] Lindheim L, Bashir M, Munzker J, Trummer C, Zachhuber V, Leber B, Horvath A, Pieber TR, Gorkiewicz G, Stadlbauer V, et al. Alterations in gut microbiome composition and barrier function are associated with reproductive and metabolic defects in women with polycystic ovary syndrome (PCOS): a pilot study[J]. PLoS One. 2017; 12(1):e168390. 10.1371/journal.pone.0168390 PMC5207627 28045919

[148] Zhou L, Ni Z, Cheng W, Yu J, Sun S, Zhai D, Yu C, Cai Z. Characteristic gut microbiota and predicted metabolic functions in women with PCOS[J]. Endocr Connect. 2020; 9(1):63–73. 31972546 10.1530/EC-19-0522 PMC6993273

[149] Liu R, Zhang C, Shi Y, Zhang F, Li L, Wang X, Ling Y, Fu H, Dong W, Shen J, et al. Dysbiosis of gut microbiota associated with clinical parameters in polycystic ovary syndrome[J]. Front Microbiol. 2017; 8:324. 28293234 10.3389/fmicb.2017.00324 PMC5328957

[150] Jobira B, Frank DN, Pyle L, Silveira LJ, Kelsey MM, Garcia-Reyes Y, Robertson CE, Ir D, Nadeau KJ, Cree-Green M. Obese adolescents with PCOS have altered biodiversity and relative abundance in gastrointestinal microbiota[J]. J Clin Endocrinol Metab. 2020; 105(6):e2134-e2144. 10.1210/clinem/dgz263 PMC7147870 31970418

[151] Ahmadi S, Jamilian M, Karamali M, Tajabadi-Ebrahimi M, Jafari P, Taghizadeh M, Memarzadeh MR, Asemi Z. Probiotic supplementation and the effects on weight loss, glycaemia and lipid profiles in women with polycystic ovary syndrome: a randomized, double-blind, placebo-controlled trial[J]. Hum Fertil (Camb). 2017; 20(4):254–261. 28142296 10.1080/14647273.2017.1283446

[152] Shoaei T, Heidari-Beni M, Tehrani HBG, Feizi A, Esmaillzadeh A, Askari G. Effects of probiotic supplementation on pancreatic beta-cell function and c-reactive protein in women with polycystic ovary syndrome: a randomized double-blind placebo-controlled clinical trial[J]. Int J Prev Med. 2015; 6:27. 25949777 10.4103/2008-7802.153866 PMC4387688

[153] Jamilian M, Mansury S, Bahmani F, Heidar Z, Amirani E, Asemi Z. The effects of probiotic and selenium co-supplementation on parameters of mental health, hormonal profiles, and biomarkers of inflammation and oxidative stress in women with polycystic ovary syndrome[J]. J Ovarian Res. 2018; 11(1):80. 30217229 10.1186/s13048-018-0457-1 PMC6137747

[154] Faure M, Bertoldo M J, Khoueiry R, Bongrani A, Brion F, Giulivi C, Dupont J, Froment P. Metformin in reproductive biology[J]. Front Endocrinol (Lausanne). 2018; 9:675. 30524372 10.3389/fendo.2018.00675 PMC6262031

[155] Practice Committee of the American Society for Reproductive Medicine. Role of metformin for ovulation induction in infertile patients with polycystic ovary syndrome (PCOS): a guideline[J]. Fertil Steril. 2017; 108(3):426–441. 28865539 10.1016/j.fertnstert.2017.06.026

[156] Xue J, Li X, Liu P, Li K, Sha L, Yang X, Zhu L, Wang Z, Dong Y, Zhang L, et al. Inulin and metformin ameliorate polycystic ovary syndrome via anti-inflammation and modulating gut microbiota in mice[J]. Endocr J. 2019; 66(10):859–870. 31270279 10.1507/endocrj.EJ18-0567

[157] Giudice LC. Clinical practice. Endometriosis[J] N Engl J Med. 2010;362:2389–2398. 20573927 10.1056/NEJMcp1000274 PMC3108065

[158] Endometriosis Pathoetiology AG. Pathophysiology: roles of vitamin A, estrogen, immunity, adipocytes, gut microbiome and melatonergic pathway on mitochondria regulation[J]. Biomol Concepts. 2019;10:133–149. 31339848 10.1515/bmc-2019-0017

[159] Ata B, Yildiz S, Turkgeldi E, Brocal VP, Dinleyici E C, Moya A, Urman B. The endobiota study: comparison of vaginal, cervical and gut microbiota between women with stage 3/4 endometriosis and healthy controls[J]. Sci Rep. 2019; 9(1):2204. 30778155 10.1038/s41598-019-39700-6 PMC6379373

[160] Jess T, Frisch M,, Jørgensen KT, Pedersen BV, Nielsen NM. Increased risk of inflammatory bowel disease in women with endometriosis: a nationwide Danish cohort study[J]. Gut. 2012; 61(9):1279–1283. 22184069 10.1136/gutjnl-2011-301095

[161] Yuan M, Li D, Zhang Z, Sun H, An M, Wang G. Endometriosis induces gut microbiota alterations in mice[J]. Hum Reprod. 2018; 33(4):607–616. 29462324 10.1093/humrep/dex372

[162] Chadchan SB, Cheng M, Parnell LA, Yin Y, Schriefer A, Mysorekar IU, Kommagani R. Antibiotic therapy with metronidazole reduces endometriosis disease progression in mice: a potential role for gut microbiota[J]. Hum Reprod. 2019; 34(6):1106–1116. 31037294 10.1093/humrep/dez041 PMC6554192

[163] M T B, Coe CL. Endometriosis is associated with an altered profile of intestinal microflora in female rhesus monkeys[J]. Hum Reprod. 2002;17:1704–1708. 12093827 10.1093/humrep/17.7.1704

[164] D H M, Marrazzo JM. The vaginal microbiome: current understanding and future directions[J]. J Infect Dis. 2016;214:S36–S41. 27449871 10.1093/infdis/jiw184 PMC4957511

[165] Ravel J, Gajer P, Abdo Z, Schneider GM, Koenig SS, McCulle SL, Karlebach S, Gorle R, Russell J, Tacket CO, et al. Vaginal microbiome of reproductive-age women[J]. Proc Natl Acad Sci U S A. 2011; 108(Suppl 1):4680–4687. 20534435 10.1073/pnas.1002611107 PMC3063603

[166] Srinivasan S, Hoffman NG, Morgan MT, Matsen FA, Fiedler TL, Hall RW, Ross FJ, McCoy CO, Bumgarner R, Marrazzo JM, et al. Bacterial communities in women with bacterial vaginosis: high resolution phylogenetic analyses reveal relationships of microbiota to clinical criteria[J]. PLoS One. 2012; 7(6):e37818. 22719852 10.1371/journal.pone.0037818 PMC3377712

[167] El Aila NA, Tency I, Claeys G, Verstraelen H, Saerens B, Santiago GL, De Backer E, Cools P, Temmerman M, Verhelst R, et al. Identification and genotyping of bacteria from paired vaginal and rectal samples from pregnant women indicates similarity between vaginal and rectal microflora[J]. BMC Infect Dis. 2009; 9:167. 19828036 10.1186/1471-2334-9-167 PMC2770471

[168] Laniewski P, Z E I, Herbst-Kralovetz MM. The microbiome and gynaecological cancer development, prevention and therapy[J]. Nat Rev Urol. 2020;17:232–250. 32071434 10.1038/s41585-020-0286-z PMC9977514

[169] Haahr T, Zacho J, Brauner M, Shathmigha K, Skov Jensen J, Humaidan P. Reproductive outcome of patients undergoing in vitro fertilisation treatment and diagnosed with bacterial vaginosis or abnormal vaginal microbiota: a systematic PRISMA review and meta-analysis[J]. BJOG. 2019; 126(2):200–207. 29469992 10.1111/1471-0528.15178

[170] Anahtar MN, Gootenberg DB, Mitchell C M, Kwon DS. Cervicovaginal microbiota and reproductive health: the virtue of simplicity[J]. Cell Host Microbe. 2018; 23(2):159–168. 29447695 10.1016/j.chom.2018.01.013

